# An Automated, Adaptive Framework for Optimizing Preprocessing Pipelines in Task-Based Functional MRI

**DOI:** 10.1371/journal.pone.0131520

**Published:** 2015-07-10

**Authors:** Nathan W. Churchill, Robyn Spring, Babak Afshin-Pour, Fan Dong, Stephen C. Strother

**Affiliations:** 1 Rotman Research Institute, Baycrest Hospital, Toronto, Ontario, Canada; 2 Department of Medical Biophysics, University of Toronto, Toronto, Ontario, Canada; 3 Institute of Medical Science, University of Toronto, Toronto, Ontario, Canada; Laureate Institute for Brain Research and The University of Oklahoma, UNITED STATES

## Abstract

BOLD fMRI is sensitive to blood-oxygenation changes correlated with brain function; however, it is limited by relatively weak signal and significant noise confounds. Many preprocessing algorithms have been developed to control noise and improve signal detection in fMRI. Although the chosen set of preprocessing and analysis steps (the “pipeline”) significantly affects signal detection, pipelines are rarely quantitatively validated in the neuroimaging literature, due to complex preprocessing interactions. This paper outlines and validates an adaptive resampling framework for evaluating and optimizing preprocessing choices by optimizing data-driven metrics of task prediction and spatial reproducibility. Compared to standard “fixed” preprocessing pipelines, this optimization approach significantly improves independent validation measures of within-subject test-retest, and between-subject activation overlap, and behavioural prediction accuracy. We demonstrate that preprocessing choices function as implicit model regularizers, and that improvements due to pipeline optimization generalize across a range of simple to complex experimental tasks and analysis models. Results are shown for brief scanning sessions (<3 minutes each), demonstrating that with pipeline optimization, it is possible to obtain reliable results and brain-behaviour correlations in relatively small datasets.

## Introduction

Blood-Oxygenation Level Dependent functional Magnetic Resonance Imaging (BOLD fMRI) is a versatile imaging modality, which is widely used in experimental neuroscience and emerging clinical applications. However, the BOLD changes linked to neuronal brain function are relatively small, and significant noise confounds are often present. The principal noise sources in fMRI are subject-dependent, including the effects of head movement and physiological processes, such as respiration and cardiac pulsation. The signal changes caused by such confounds are highly variable between subjects, and even across scanning sessions for a single subject, with complex spatial and temporal structure. This limits our ability to reliably detect neuronal-linked BOLD signals with adequate power, especially for complex task paradigms and studies of clinical, aging and child populations [1–4]. Consequently, there is much debate concerning the reproducibility, validity and power of published fMRI measurements [5–10]. The resulting low power and low reliability of fMRI also limits our ability to measure brain-behaviour relationships, which is a key goal of many fMRI studies.

To control noise and improve signal detection, a variety of image preprocessing algorithms have been developed, from generalized techniques (e.g. spatial smoothing of brain voxels) to artifact-specific correction (e.g. motion correction algorithms). Over the past two decades, it has been established that the chosen set of preprocessing steps and analysis model (the “pipeline”) significantly impacts fMRI results [11–22]. Nonetheless, most fMRI literature has not emphasized the quantitative validation of preprocessing choices, implicitly assuming that analysis results are insensitive to them, or that the widely-used, open-source preprocessing packages produce near-optimal results. This has led to inconsistent, often under- and un-reported pipeline methodologies [23–25], and sub-optimal signal detection in fMRI experiments, all of which contribute potential bias and unwanted methodological noise in the quest to characterize brain function and brain-behaviour relationships.

Some of the issues with sub-optimal signal detection may be improved by making well-motivated choices in how fMRI data are preprocessed [21,22]. For example: there are significant differences in the robustness of different motion correction algorithms [17]; the impact of residual motion correction techniques depends largely on the choice of experimental design and task contrast [26,27]; physiological noise corrections may significantly reduce differences between analysis models [11]; and the order in which preprocessing steps are performed has a significant impact on their efficacy [28,29].

Nonetheless, choosing the optimal sequence of preprocessing steps is a daunting task; while it is important to make sensible pipeline choices, many algorithms have been published, and it quickly becomes non-trivial to account for the many possible interactions between experimental task design, preprocessing and analysis algorithms. Some advocate a conservative approach, using a fixed, standardized pipeline to control all anticipated noise confounds [9,30]. This strategy limits pipeline flexibility and reduces power, but provides strong control against false-positive activations. Overly-flexible preprocessing selection is a significant issue if unconstrained, or if pipelines are chosen to maximize the significance of findings, leading to highly biased results [31].

As an alternative, we show that flexible, adaptive pipeline optimization is a powerful tool for improving signal detection in fMRI, if we select preprocessing steps that optimize the statistical analysis criteria of prediction accuracy (*P*) and spatial reproducibility (*R*). In this paper, we propose an automated, adaptive framework, which optimizes the preprocessing of individual subject task runs, by identifying the pipeline that maximizes (*P*,*R*) metrics. It is based on the NPAIRS resampling framework of [32], and constitutes a significant extension of previous work on pipeline optimization [13–14,20–22]. This framework is an alternative to standard preprocessing methods in fMRI literature, which are usually based on subjective visual assessments of data quality; these are time-consuming to evaluate and may lead to biased, non-replicable results.

**Fig 1:**
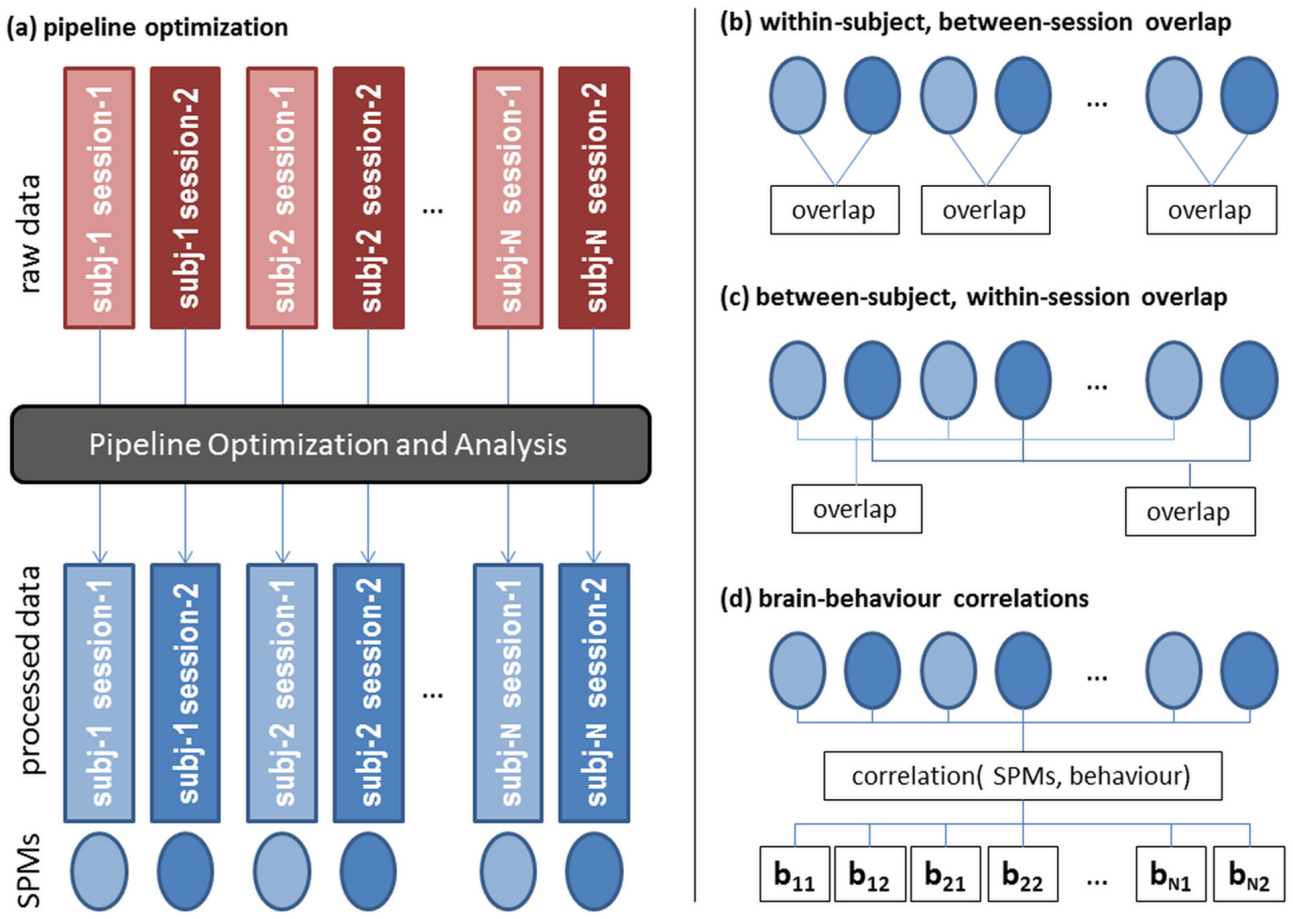
The pipeline optimization and validation procedure of this paper. This schematically represents the approach to pipeline optimization, and independent validation measures used to test the quality of optimized results. (a) for all subjects and each of the three tasks, we have raw data from two scanning sessions (i.e. test and retest) in red. For each task run and session, we identify the optimal pipeline based on measures of prediction and reproducibility (P, R), and output optimally preprocessed data (blue) and an analysis SPM. (b-d) cross-validation measures, computed on SPMs of independently-optimized datasets. (b) activation overlap between SPMs of the two scanning sessions, within each subject. (c) activation overlap between subject SPMs, within a single scanning session. (d) correlation between SPM activity and independent behavioural measures (b_ij_ = subject i, session j), measured via Partial Least Squares analysis.

This paper establishes the framework used to preprocess individual scanning runs, along with independent validation measures to evaluate the effects of pipeline optimization, which are summarized in Fig 1. Preprocessing steps are selected to independently maximize (*P*, *R*) metrics and the resulting statistical parametric maps (SPMs) for individual task runs within scanning sessions (Fig 1a; separate light and dark blue data sets, and their SPMs). We validate this approach by measuring the reliability of SPM activation patterns from these independently-optimized task runs, including within-subject, between-session comparisons (i.e. test-retest reliability; Fig 1b), and between-subject, within-session comparisons (i.e. group-level reliability; Fig 1c). In addition, we measure the correlation between the SPM activation patterns and behavioural metrics measured and tested completely independently of our pipeline optimization procedures (i.e. brain-behaviour relationships; Fig 1d). Because we are comparing independently-optimized runs, these measures avoid issues of circularity when quantifying model performance [31]. We demonstrate that our pipeline optimization framework significantly improves all three independent validation measures, across multiple tasks and analysis models.

## Materials and Methods

In this section, we first describe the different task datasets used to evaluate pipeline optimization (*Experimental Data*). We then establish the set of preprocessing and analysis options included in the pipeline framework (*Preprocessing Pipeline Steps; Analysis Models*). Afterwards, we define metrics of prediction and reproducibility, which are used to quantify pipeline performance (*Optimization Metrics*), and the different approaches to pipeline selection that are compared in this paper (*Pipeline Optimization Approaches*). Afterwards, we measure the effects of pipeline optimization on performance metrics and spatial brain patterns (*The Effects of Pipeline Optimization*), along with independent validation measures of spatial reliability (*Validation 1*: *Spatial Reliability of Independent Sessions*) and brain-behaviour correlations (*Validation 2*: *Estimating Brain-Behaviour Correlations*). The fMRI pipeline results and behavioural measures are deposited at figshare.com (doi: 10.6084/m9.figshare.1299085).

### Experimental Data

We performed pipeline analyses on data from a cognitive task battery, designed for clinical implementation in the assessment of stroke and vascular-cognitive impairment. We collected data from 27 young, healthy volunteers (15 female, ages 21–33 yrs, median 25 yrs), and we acquired retest session data from 20 out of 27 volunteers (12 female, ages 22–33 yrs, median 25 yrs) at a median 6 months after the initial testing session (range 2–23 months). Participants were confirmed right-handed with the Edinburgh Handedness Inventory [33], and screened for cognitive and neurological deficits, by self-report and the Mini-Mental Status Examination [34]. All participants gave written informed consent for their participation and the experiment was conducted in the Rotman Research Institute, Baycrest Hospital, with the approval of the Baycrest Research Ethics Board.

BOLD fMRI data were acquired on a 3T MR scanner (MAGNETOM Tim Trio, VB15A software; Siemens AG, Erlangen, Germany), with a 12-channel head coil. A T1-contrast anatomical scan was obtained (oblique-axial 3D MPRAGE, 2.63/2000/1100 ms TE/TR/TI, 9° FA, 256 X 192 matrix, 160 slices per volume, voxel dimensions 1x1x1 mm^3^), followed by BOLD fMRI (2D GE-EPI, 30/2000 ms TE/TR, 70° FA, 64x64 matrix, 30 slices per volume, voxel dimensions 3.125x3.125x5 mm^3^). During scanning, we also measured cardiac and breathing rates via photoplethysmograph and pneumatic belt, respectively.

For both test and retest sessions, participants received a 15 minute orientation in an MRI simulator, and performed two runs of each task in the scanner, separated by approximately 10 minutes of other behavioural tests. The tasks in the battery included an initial encoding task (ENC), followed by a block-design adaptation of the Trail-Making Test (**TMT**), a block-design, forced-choice recognition tasks (**REC**) of the encoded line drawings, and a rapid event-related Sustained Attention to Response Task (**SART**). The tasks were relatively brief (<3 minutes each), and involved a range of different cognitive contrasts, to explore the effects of pipeline optimization under different experimental designs. We focused on pipeline optimization within relatively brief runs (i.e. within each of the two task runs per testing session), to demonstrate that we can obtain reliable measures in small, complex task datasets if preprocessing is optimized. All tasks were performed in the fMRI scanner using an fMRI-compatible response tablet [35], and presented to subjects in a fixed ordering of ENC, TMT, REC, SART to ensure a constant delay between encoding and recognition tasks. The encoding of line drawn objects involved overt naming and will be addressed in future work.

#### Recognition (REC)

Alternating scanning task and control blocks of 24 s were presented 4 times, for a total task scanning time per subject of 192 s. During the task blocks, participants were presented with a previously encoded figure side-by-side with two other figures (semantic and perceptual foils) on a projection screen every 3 s, and were asked to touch the location of the original figure on the tablet. Figures were line drawn objects from the Boston Naming test [36]. During control blocks, participants touched a fixation cross presented at random intervals of 1–3 s. We analyzed the contrast between recall and control tasks, as a robust block-design contrast.

#### Trail-Making Test (TMT)

The task was similar to the widely used clinical version [37], consisting of stimulus types: *TaskA*, in which numbers 1–14 are pseudo-randomly displayed on a viewing screen, and *TaskB*, in which numbers 1–7 and letters A-G are displayed. Subjects used the tablet to draw a line connecting items in sequence (1-2-3-4-…) or (1-A-2-B-…), connecting as many as possible for a 20s block interval, while maintaining accuracy. A *Control* stimulus was presented after each block, in which participants traced a line from the center of the screen to a dot (randomly placed at a fixed radius from the center of the screen) repeated 10 times. For a single run, each participant performed a 4-block, 40-scan epoch of *TaskA*–*Control*-*TaskB*-*Control* twice. We analyzed the contrast between Task B and Task A conditions, as a relatively subtle block-design contrast of brain states.

#### Sustained Attention to Response Task (SART)

This task was presented as a fast event-related GO-NOGO design [38]. The set of integers 1–9 were presented in random order on the screen, followed by a masking image. Stimuli were presented for 250 ms, while the mask was shown for a randomized inter-stimulus interval, of mean 1250 ± 210 ms. Participants were asked to respond to all integers except ‘3’ (the NOGO stimulus) using the MRI-compatible writing tablet, by touching the stylus to the tablet surface. A single run consisted of 100 presented digits, with 75 GO stimuli and 25 NOGO stimuli, in randomized order, with 76 scans per run. We estimated the main haemodynamic response associated with GO stimulus in a 9-TR time window, as a representative event-related design.

### Preprocessing Pipeline Steps

The proposed fMRI pipeline is an automated framework, which is used to measure the effects of different preprocessing choices on signal detection in fMRI data. This framework can test any combination of pre-existing or new preprocessing algorithms, and determine the set of preprocessing choices that optimizes signal detection based on our prediction and reproducibility metrics. Here, we establish a 13-step pipeline, with a focus on optimizing a comprehensive set of 9 steps. All of these pipeline steps are either widely used in the fMRI literature, or have a significant impact on task performance, based on prior studies. The pipeline steps are listed in Table 1, in the order in which they were applied, along with the options tested for each step. For the purposes of this report we did not attempt to test the much more computationally intensive possibilities of different orderings of the steps.

**Table 1:** List of pipeline steps, and choices tested for each step. Steps that are varied during each subject’s pipeline optimization are in **bold**, and other steps are held fixed. We tested pipeline optimization for fixed analysis models: Gaussian Naïve Bayes (GNB; univariate) and Canonical Variates Analysis (CVA; multivariate). CVA analysis is performed using 1 to k Principal Components (PCs), where we vary **k = 1 to 10**.

PIPELINE STEPS	CHOICES
1. Estimate minimum-displacement brain volume	ON
**2. Rigid-body motion correction**	**OFF** / **ON**
**3. Censoring of outlier brain volumes**	**OFF** / **ON**
**4. Physiological correction; external physiological measures (RETROICOR)**	**OFF** / **ON**
**5. Slice-timing correction**	**OFF** / **ON**
6. Spatial smoothing	6mm FWHM
7. Subject-specific non-neuronal tissue mask	ON
**8. Temporal detrending**	**orders 0 to 5**
**9. Motion parameter regression**	**OFF** / **ON**
**10. Global signal regression using Principal Component Analysis (PCA)**	**OFF** / **ON**
**11. Including task design as a regressor**	**OFF** / **ON**
**12. Physiological correction; multivariate data-driven model (PHYCAA+)**	**OFF** / **ON**
13. Analysis model: univariate (GNB) or multivariate (CVA)*	GNB or CVA

When processing an fMRI dataset, the pipeline consists of the following sequence of 13 steps. The 9 steps in **bold** are tested during pipeline optimization, while other steps are fixed, and applied to all datasets. Preprocessing steps (2–6) are based on utilities in the widely-used AFNI package (Analysis of Functional Neuroimaging; afni.nimh.nih.gov/afni); all other steps were developed in-house, and developed in Matlab (MATLAB and Statistics Toolbox Release 2012b, The MathWorks, Inc., Natick, MA).

1. Estimate minimum-displacement brain volume: identify the volume with minimum head displacement in the scanning run, which had minimum Euclidean distance from the median coordinates in Principal Component (PCA) space of the 4D data set. This is used as a reference for Motion Correction (step 2) to minimize the average distance that motion alignment displaces each brain volume, as the accuracy of Motion Correction decreases with distance from the reference volume [39].
2. **Motion correction [OFF/ON]**: use the AFNI *3dvolreg* algorithm to transform each image to the volume with minimum estimated displacement, to correct for rigid-body head motion. This step is tested in the pipeline, as its effects vary by dataset: it reduces motion artifact, particularly younger and older groups, and clinical datasets [1,4,40], but may produce biased results in cases of large BOLD response and relatively small head movements [41].
3. **Censoring of outlier brain volumes [OFF/ON]**: remove outlier timepoints that are caused by abrupt head motion, and replace them by interpolating from adjacent volumes (algorithm is fully described in S1 Text; code available at: www.nitrc.org/projects/spikecor_fmri). The censoring step is a robust alternative to typical “scrubbing” algorithms [42–43], which is fully automated, and does not create discontinuities in the data [44]. There have been no major studies of censoring in fMRI task data, and thus its impact and importance as a preprocessing step is largely unknown.
4. **Physiological correction; external physiological measures [ON/OFF]**: apply RETROICOR [45], using AFNI’s *3dretroicor* software. This parametric model uses external measures of respiration and heartbeat. A 2^nd^-order Fourier series was used to fit voxel time-courses, relative to the phase of cardiac and respiratory cycles. This step is optimized, as its impact on signal detection has been shown to vary as a function of subject and dataset [21–22].
5. **Slice-timing correction [OFF/ON]**: correct for timing offsets between axial slices due to EPI acquisition, by using AFNI’s 3dTshift with Fourier interpolation to resample the voxel time-courses. For event-related data that require estimation of the temporal haemodynamic response (e.g. SART), this step is fixed **ON**. For block designs, this step is tested during pipeline optimization; while Sladky et al. [46] showed that slice-timing correction improves detection power in block designs, we have observed subject-dependent effects of including this step [47], which may be due to interactions with critically-sampled physiological noise not removed by a previous RETROICOR step. Our results show a significant impact on prediction and reproducibility metrics of slice timing with block designs for some subjects.
6. Spatial smoothing: the brain volumes are spatially smoothed with a 3D isotropic Gaussian kernel, using the AFNI *3dmerge* algorithm. For current results, we use a fixed scale of FWHM = 6mm; this parameter may be varied in future studies. For example, the size and “focalness” of activations vary by task [48], and the smoothing scale may be chosen to optimize the detection of these brain regions [14]. Interactions with spatial smoothing scale are testable in our framework, but beyond the scope of the current paper, which is focused on the optimization of temporal preprocessing choices.
7. Subject-specific non-neuronal tissue mask: generate a data-driven mask of non-neuronal tissues (vasculature, sinuses and ventricles) that should be excluded prior to analysis. Otherwise, these voxels produce false-positive activations, and biased estimates of spatial reproducibility. This step uses the PHYCAA+ algorithm [49] to estimate subject-specific masks, to account for inter-subject differences in vasculature.
8. **Temporal detrending [order 0 to 5]**: regress out low-frequency fluctuations from fMRI data, by fitting a Legendre polynomial of order *N* in a General Linear Model (GLM, which also includes steps 9 to 11). The algorithm tests detrending with an *N* ^th^-order polynomial, for n = 0 to 5. Detrending provides non-specific noise correction, including head motion, scanner drift, and physiological noise [50]. Different detrending models are tested, as the optimal order varies as a function of subject and task design [12,21–22].
9. **Motion parameter regression [OFF/ON]**: perform PCA on the motion parameter estimates (output from *3dvolreg* in step 2), and identify the 1-k PCs that account for >85% of motion variance. These components are regressed from the data in a GLM model, which includes steps 8, 10–11. This step is tested in the pipeline, as its effects vary by dataset: it controls residual motion artifact [4,41,50], but it may also reduce experimental power, particularly in cases of large BOLD response and low head motion [21, 26–27].
10. **Global signal regression using PCA [OFF/ON]**: perform PCA on the fMRI data and regress out PC#1 time-series, which tends to be highly correlated with global signal effects, as part of a GLM including steps 8–9, 11. This approach minimizes the distortion of signal independent of global effects, unlike simple regression of mean BOLD signal [51]. The exact mechanism underlying global modulation remains unclear, but it may constitute physiological noise [52], neuronal response [53], or a mixture of both. The magnitude of global signal expression appears to be subject-dependent [54,55], indicating the importance of adaptively estimating it across subjects.
11. **Including task design as a regressor [OFF/ON]**: convolve the task paradigm with AFNI’s standard ‘SPMG1’ HRF function (afni.nimh.nih.gov/pub/dist/doc/program_help/3dDeconvolve.html). This regressor is included in the GLM model with steps 8–10. When these nuisance regressors are correlated with the task paradigm, step 11 protects against over-estimation of noise variance, and over-regression of task-related signal. This step is tested in the pipeline: although it controls against over-regression of task-related signal, the most robust BOLD response may be only weakly correlated with the task paradigm [56], and this step may over-constrain subsequent analyses.
12. **Physiological correction; multivariate data-driven model [OFF/ON]**: use the multivariate data-driven PHYCAA+ model [49] (code available at: www.nitrc.org/projects/phycaa_plus) to identify physiological noise components in the data, which are regressed out from the fMRI data. It has been previously demonstrated that this step significantly improves the prediction and reproducibility of fMRI task analyses.
13. Analysis: for each combination of pipeline steps, the preprocessed data are analyzed in the NPAIRS split-half framework [32] previously described in [22]. We test pipeline optimization for two predictive analysis models: univariate (Gaussian Naïve Bayes) and multivariate (Canonical Variates Analysis), discussed in the next section. For multivariate analyses, we perform **PCA subspace estimation [dimensionalities *k* = 1 to 10]**, by transforming each data split into a reduced principal component subspace, of PCs 1-*k*. This may be thought of as a PCA denoising step, in the preprocessing for our multivariate model. We then analyze each split, producing metrics of (P) Prediction accuracy and (R) spatial Reproducibility of the activation maps, for the pipeline data.

Steps 8–10 are regressed as nuisance covariates in a General Linear Model (GLM), and Step 11 includes the task paradigm in the same GLM design matrix, to protect against over-regression of task-related BOLD signal. From this list of choices, we can test a large number of different preprocessing pipelines by turning each of the 9 optional steps off and on. The total number of tested pipelines per subject is 2^8^x6 = 1,536 (Gaussian Naïve Bayes analysis) and 2^8^x6x10 = 15,360 (Canonical Variates Analysis).

### Analysis Models

The analysis models that have been developed for fMRI task data can be broadly categorized as either univariate or multivariate. Univariate models assume brain voxels are independent random mixtures of signal and noise. This is a simplification, as brain regions have significant functional correlations [57–59]; nonetheless, it provides a well-posed model of brain activity that is easy to interpret. Multivariate models account for covariance between brain regions, identifying regions that fluctuate coherently in response to stimuli; they are effective when individual voxels are noisy, but co-vary strongly. In this paper, we perform pipeline optimization for representative univariate and multivariate analysis models. Both are predictive models that use a *training* dataset to construct a model of brain activity, and use this model to predict the experimental condition of independent *test* data.

#### Univariate analysis

We employed a Gaussian Naïve Bayes model (GNB; a predictive GLM) in order to perform classification on independent test data. It is one of the most widely used predictive models in fMRI literature [60], and measures the joint posterior probability of all brain voxels in test data, along with a sensitivity map of activated voxels [61,62]. For REC and TMT, we classify test data from two task conditions (2-class prediction). For SART, we estimate an HRF in a 9-TR time window (9-class prediction, where each time-lag is a class). See *Optimization Metrics* for further prediction details, along with the appendix of [49].

#### Multivariate analysis

We employed Canonical Variates Analysis (CVA), which has been used in numerous studies [4,13–15,20–22,62], and estimates a multivariate Gaussian model for fMRI task conditions. CVA is highly flexible, able to analyze block and event-related data, and generalizes to an arbitrary number of task conditions. For 2-condition REC and TMT tasks, it is equivalent to a linear discriminant, and obtains one brain eigenimage. For SART data, analyzed in a 9-TR time window, we optimize the first eigenimage, which reflects the primary HRF. As with GNB, we perform 2-class prediction for REC and TMT, and 9-class prediction for SART.

### Optimization Metrics

Our goal is to identify the optimal preprocessing pipelines, which maximize the detection of neuronal-linked BOLD response and minimize noise confounds. A major challenge is to quantify the impact of preprocessing choices on analysis results in fMRI, in the absence of a “ground truth”. The BOLD amplitude and regions of brain activation vary across subjects and sessions; therefore, there is no single generalized BOLD response to a stimulus. Moreover, we may not know the expected pattern of brain activation for novel task paradigms. Simulations, though instructive, provide limited information on pipeline effects. It remains an ongoing challenge to simulate the complexity of brain networks, and current models do not contain the same information content that is present in real data [63]. Two alternative metrics are used to measure pipeline effects in experimental data: the prediction accuracy of the analysis models, and the spatial reproducibility of brain maps, computed in the split-half NPAIRS framework [32]; these metrics are briefly summarized, but refer to [13–15,21–22,32] for further details.

The split-half approach is used to independently optimize every task run, i.e. it is applied separately to each of the 6 task runs (2 runs per task type) in each testing session. For a single continuous task run, this preprocessed dataset is split (in time) into two halves, which are preprocessed and analyzed independently. We use the independent analysis results to compute **Prediction** (*P*), where a classifier (analysis) model is built on *training* data in a single split-half, and we measure its ability to correctly predict the experimental condition of scans from an independent *test* dataset, i.e. the other split-half. This is given by the average posterior probability *P* that test scans are correctly assigned to the true experimental condition. As a probability measure, *P* takes values in the range [0,1] where *P* = 1 indicates perfect prediction. Prediction quantifies how well our analysis model generalizes to new fMRI data.

We also compute **Reproducibility** (*R*), which measures how stable the activation patterns are across independent data split-halves. We obtain *R* by the Pearson correlation between pairwise voxel values of the two brain maps. This metric can take values in the range [–1,1], with *R* = 1 indicating a perfectly reproducible brain map. The global Signal-to-Noise Ratio (*gSNR*) of BOLD response can be computed based from reproducibility [62], by the equation $gSNR=\sqrt{2R/\left(1-R\right)}\;$ We also use the two split-half brain maps to estimate a reproducible Z-scored Statistical Parametric Map (SPM), for which [32] provides further details.

For every individual run, we apply each of the 1,536 (or 15,360) preprocessing combinations, analyze each pipeline, and obtain (*P*, *R*) measures. We then select the pipelines that optimize (*P*, *R*) values. Although prediction and reproducibility are important goals for any neuroscientific experiment, it is rarely possible to simultaneously optimize both metrics. This is due to *P* and *R* representing important trade-offs in model parameterization, making it generally undesirable to strictly optimize one metric [56]. Models that optimize *R* have more stable brain patterns, but are often less sensitive to stimulus-coupled brain response (i.e. they exhibit weak prediction). For example, an analysis model that ignores data input and generates a fixed brain pattern will be perfectly reproducible (*R* = 1), but with no ability to predict brain state. Conversely, models with optimized *P* are highly predictive of stimulus condition, but tend to extract non-reproducible brain patterns. For example, a model that only selects a small number of the highly task-coupled brain voxels may be highly predictive of class structure (*P* ≈ 1), but will have low reproducibility, as the selected voxels vary between splits due to random signal/noise variations. Standard analysis models and experimental data rarely produce such extreme results. However, the choice of optimization criteria significantly alters results, potentially identifying different or partial brain networks with varying signal strengths and spatial extents [15,56,62]. This paper therefore focuses on pipeline optimization by minimizing Euclidean distance *D*(*P*, *R*), relative to perfect model performance (*P* = 1, *R* = 1). The joint optimization of (*P*, *R*) provides a compromise between the two model parameterizations, which can be used to select an optimal pipeline [13,20].

### Pipeline Optimization Approaches

**Fig 2:**
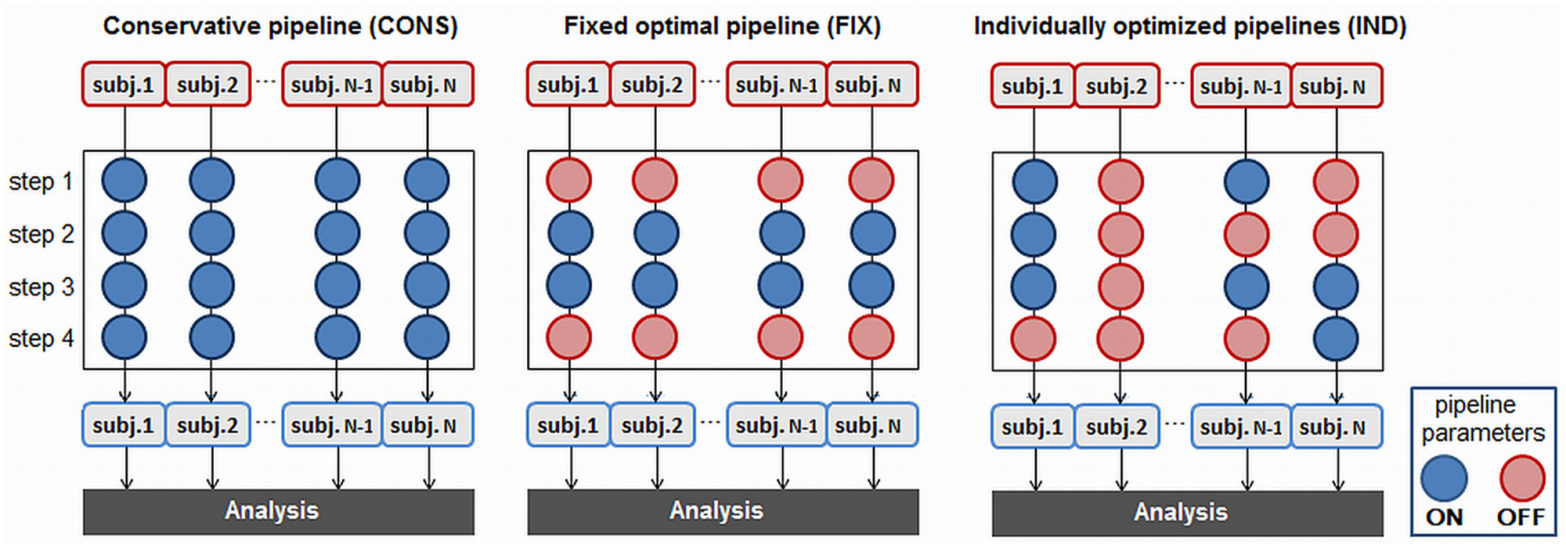
Different approaches to optimizing preprocessing pipelines. This toy example depicts N subjects, with 4 preprocessing pipeline steps; each step may be either applied to data (ON) or not applied (OFF). A standard conservative approach (CONS) applies all commonly-used noise correction steps to fMRI data. A fixed optimal pipeline (FIX) applies the single set of pipeline steps that optimizes average prediction and reproducibility (P,R) across subjects. Individual optimization (IND) selects the combination of pipeline choices, specific to each subject and session, which maximizes prediction and reproducibility (P, R). To see the preprocessing choices for optimized pipelines in our current results, refer to S1 and S2 Tables.

This paper compares three different approaches to pipeline selection, which are described schematically in Fig 2. S1 and S2 Tables list the fraction of subjects optimized with each preprocessing step, as a function of optimization pipeline. For the current results, all steps are optimized except pipeline steps (1, 6, 7), which are fixed ON. Step 1 (selection of minimum displacement volume) is fixed, so that we can compare the same MOTCOR procedure across all pipelines; Step 6 (spatial smoothing) is fixed in order to compare pipelines activations at a consistent spatial scale; Step 7 (masking non-neuronal brain voxels) is required to compare a consistent set of brain voxels across all pipelines. Note that because of Steps 1 and 7, the CONS pipeline will be more optimized than is standard literature practice.

#### Conservative pipeline (CONS)

This applies the full set of preprocessing steps that are widely used in fMRI preprocessing (steps 2–5, 8, 9): motion correction, outlier censoring, RETROICOR, slice-timing correction, motion parameter regression, and linear detrending (chosen by AFNI's heuristic criterion; afni.nimh.nih.gov/pub/dist/doc/program_help/3dDeconvolve.html). This gives strong control over potential fMRI noise sources, and provides an example of a standard literature preprocessing pipeline, which we compare against our adaptive optimization methods. For this pipeline, we apply the same set to all subjects and experimental tasks.

#### Fixed optimization (FIX)

For each task, we select a single, fixed set of pipeline choices across subjects, that give smallest average *D*(*P*, *R*). We use a non-parametric procedure established in [21–22] to identify the optimal fixed pipelines: for *M* pipelines and *S* subjects, (1) rank the pipelines 1-*M* for each subject, with lower rank indicating better pipeline performance; (2) compute the median ranking of each pipeline, across subjects; (3) select the pipeline with lowest median ranking, as our optimal FIX choice. We can perform further statistical testing to determine whether fixed pipeline choice has a consistent, significant impact on *D*(*P*, *R*); Churchill et al. [22] provide in-depth discussion of fixed pipeline testing. The FIX pipeline is the single fixed set of preprocessing choices with highest median (*P*, *R*) across subjects. All other fixed pipelines will have comparable or lower median (*P*, *R*) and *gSNR* values.

#### Individual pipeline optimization (IND)

For each subject, session, run and task, we identify the pipeline combination that maximizes *R* (IND-R), maximizes *P* (IND-P) or minimizes *D*(*P*, *R*) (IND-D). For IND optimization, we require an additional step to account for task-coupled motion, which generates artifact that is task-correlated and reproducible, and thus not controlled by optimizing (*P*, *R*) metrics. We used the quantitative procedure established in [22] to reject pipelines corrupted with motion artifact when optimizing; the procedure is described in S2 Text.

### The Effects of Pipeline Optimization

We computed the mean (*P*, *gSNR*) values, within each individual task run and analysis model of the first test session. In each case, the mean is computed across all (27 subjects) x (2 runs per task) = 54 datasets, along with the ± 1 Standard Deviation ellipse, enclosing ~68% of data points. We also measured the average correlation between all optimised pipeline/analysis model SPMs, for each experimental task. For each of the 54 datasets, we compute the 6x6 correlation matrix between brain maps of each pipeline and analysis model combination. We then computed the average of all of these correlation matrices.

Finally, for each pipeline optimization approach, we produced a Z-scored plot of the first PC eigenimage, computed over all 54 SPMs. This is shown for representative TMT data and the CVA analysis model (see S3 Text for the estimation procedure). The Z-scored eigenimages depict the brain pattern that expresses the greatest variance across all subject SPMs, for the full set of (27 subjects) x (2 runs per session) = 54 datasets. The Z-scores in these maps quantify statistical reproducibility of the eigenimage values.

### Validation 1: Spatial Reliability of Independent Sessions

We used activation overlap to test whether datasets with independently optimized IND pipelines show greater reliability of brain regions compared to CONS. Because IND pipeline optimization is performed entirely within individual scanning runs (i.e. no information is shared between subjects, between repeated task runs within a session, or between test-retest sessions), we can independently compare SPMs between scanning sessions and between subjects, without any issues of circularity in model validation.

Activation overlap is widely used in the fMRI literature to measure the reliability of significantly active brain regions [8]. For each SPM, we identified active voxels at a False-Discovery Rate (FDR) = 0.05 threshold, to correct for multiple comparisons. We then measured pairwise overlap using the Jaccard index, (intersection of active voxels)/(union of active voxels). We measured both within-subject, between-session overlap, and within-session, between-subject overlap. For all 27 subjects, we have a test session with 2 runs per task. For 20 of these subjects, we also have a retest session with 2 runs per task, acquired a median of 6 months after the test session. Overlap measures are computed as follows:

#### Within-subject, between-session testing

For each task, we measured the pairwise overlap between (1) run-1 (test) vs. run-1 (retest) sessions, and (2) run-2 (test) vs. run-2 (retest), for all 20 subjects with retest data. We chose to compare test-retest overlap within runs, in order to avoid possible confounds due to non-stationary learning and habituation effects between run-1 and run-2. After computing all pairwise overlaps, this produced (2 runs) x (20 subjects) = 40 independent measures of overlap, for each task.

#### Between-subject, within-session testing

Using only data within a single task run and test session, we measured mean overlap of each subject with all others in the group; this was performed separately for the two task runs in each session, to minimize non-stationary in BOLD response as a function of run or session. For the first test session, this produced (27 mean overlap estimates)x(2 runs) = 54 overlap measures. For the second retest session, this produced (20 mean overlap estimates)x(2 runs) = 40 overlap measures. For each task, this produces 94 mean inter-subject overlap values total.

We then plotted the distribution of activation overlap values for CONS vs. IND pipelines, for each experimental task and analysis model, including the mean over all 40 (within-subject) or 94 (between-subject) overlap measures, and the ± 1 Standard Deviation ellipse, enclosing ~68% of data points.

### Validation 2: Estimating Brain-Behaviour Correlations

One of the major goals of fMRI is to link brain function with behaviour. Therefore, an important test of pipeline optimization is whether it improves the reliability and generalizability of brain-behaviour correlations across subjects, which is independent of our (*P*, *R*) pipeline optimization criteria applied within subject, task and session. It is important to note that this test is unrelated to the spatial reliability of SPMs discussed in *Validation 1*: *Spatial Reliability of Independent Sessions*. We may obtain a highly reliable SPM pattern across subjects, but if the magnitude of activation is unrelated to task performance, this produces low brain-behaviour correlations. Conversely, the SPM patterns may be spatially sparse and generally unreliable across subjects, but with a subset of brain regions where activation is highly correlated with task performance. Thus, it is important to understand how pipeline choices affect both of these validation measures.

To measure brain-behaviour relationships, we performed Partial Least Squares (PLS) analysis of the optimized pipeline SPMs against behavioural metrics. The PLS model is widely used in fMRI [64]. It estimates the spatial brain map of greatest covariance with a behavioural measure of interest. Behavioural PLS was performed in a split-half estimation framework [65] (see S4 Text for algorithm details). This model uses a resampling approach similar to the one in *Optimization Metrics*, producing (1) a reproducible Z-scored map of brain regions showing greatest covariance with behavioural performance, (2) the global Signal-to-Noise Ratio of the behavioural brain pattern (*gSNR*_behav_), and (3) an unbiased measure of multivariate brain-behaviour correlation (ρ_behav_). We compute median *gSNR*_behav_ and ρ_behav_ values, and average Z-scored brain map, over 100 resampling iterations. This behavioural PLS analysis is performed separately for both CONS and IND pipeline SPMs.

We use these results to test whether there is a significant, reliable difference between median (*gSNR*_behav_, ρ_behav_) for IND vs. CONS pipelines, using Bootstrap resampling. We perform sampling with replacement on the 100 split-half estimates, compute the median (*gSNR*_behav_, ρ_behav_) for both pipelines, and then measure Δ*gSNR* = (*gSNR*_IND_−*gSNR*_CONS_) and Δρ = (ρ_IND_− ρ_CONS_). This is repeated for 1000 iterations, and we measure the fraction of resamples in which Δ*gSNR* >0 and Δρ >0 (i.e. 100,000 resamples total). This provides empirical significance estimates on the difference between IND and CONS pipelines. We performed behavioural PLS analysis with significance testing, for the three different tasks and two different analysis models.

#### Behavioural measures

The behavioural metrics analyzed for each task are listed below. In order to maximize power of the PLS analyses, we examined all (27 test + 20 retest subjects) x (2 sessions per run) = 94 data points for each task.

- Recognition (REC): we analyzed the difference in mean reaction time (RT) for (task—control), measured in milliseconds. We averaged mean RTs across all task onsets, for a given task session.
- Trail-Making Test (TMT): we analyzed the difference in average inter-item speed for (TrailsA—TrailsB), measured in m/s. We measured the time interval between completion of subsequent items, divided by the total distance traversed by the cursor, and averaged across all blocks for a given task session.
- Sustained Attention to Response Task (SART): we analyzed the accuracy of task performance, measured as the fraction of correct GO-condition responses (button press after viewing stimuli) per run.

Multivariate behavioural analysis is highly sensitive to outlier data, an issue that increases in smaller sample sizes and complex, heterogeneous tasks. For all pipelines, we performed two outlier tests prior to PLS analysis: one to identify behavioural outliers and one to identify outliers in fMRI data. Behavioural outlier subjects were identified if either (a) mean reaction time was less than 100 ms, (b) mean accuracy was less than 50%, or (c) mean RT was shorter for the more difficult task (recall task for REC, and TaskB for TMT; this indicates that learning effects may predominate in the task). The fMRI outlier estimation process is a multivariate procedure based on the RV coefficient, defined in S5 Text. Data sets that were outliers in either behaviour or fMRI data were discarded, and PLS analysis performed on the remaining data points. S3 Table provides a summary of the discarded outliers for each task; for all tasks and analysis models, we identified between 9 and 12 outlier runs (out of 94 datasets).

## Results

In this section, we first demonstrate the effects of pipeline optimization on performance metrics and spatial brain patterns (*The Effects of Pipeline Optimization*), then we show the impact of pipeline optimization for independent validation measures of spatial reliability (*Validation 1*: *Spatial Reliability of Independent Sessions*) and brain-behaviour correlations (*Validation 2*: *Estimating Brain-Behaviour Correlations*). The results are shown for three different experimental tasks: the block-design **REC** task, the more complex block-design **TMT** task, and the fast event-related **SART** task. We also analyzed all fMRI data using both univariate **GNB** (a predictive GLM) and multivariate **CVA** models, to show that pipeline optimization effects generalize across models.

### The Effects of Pipeline Optimization

**Fig 3:**
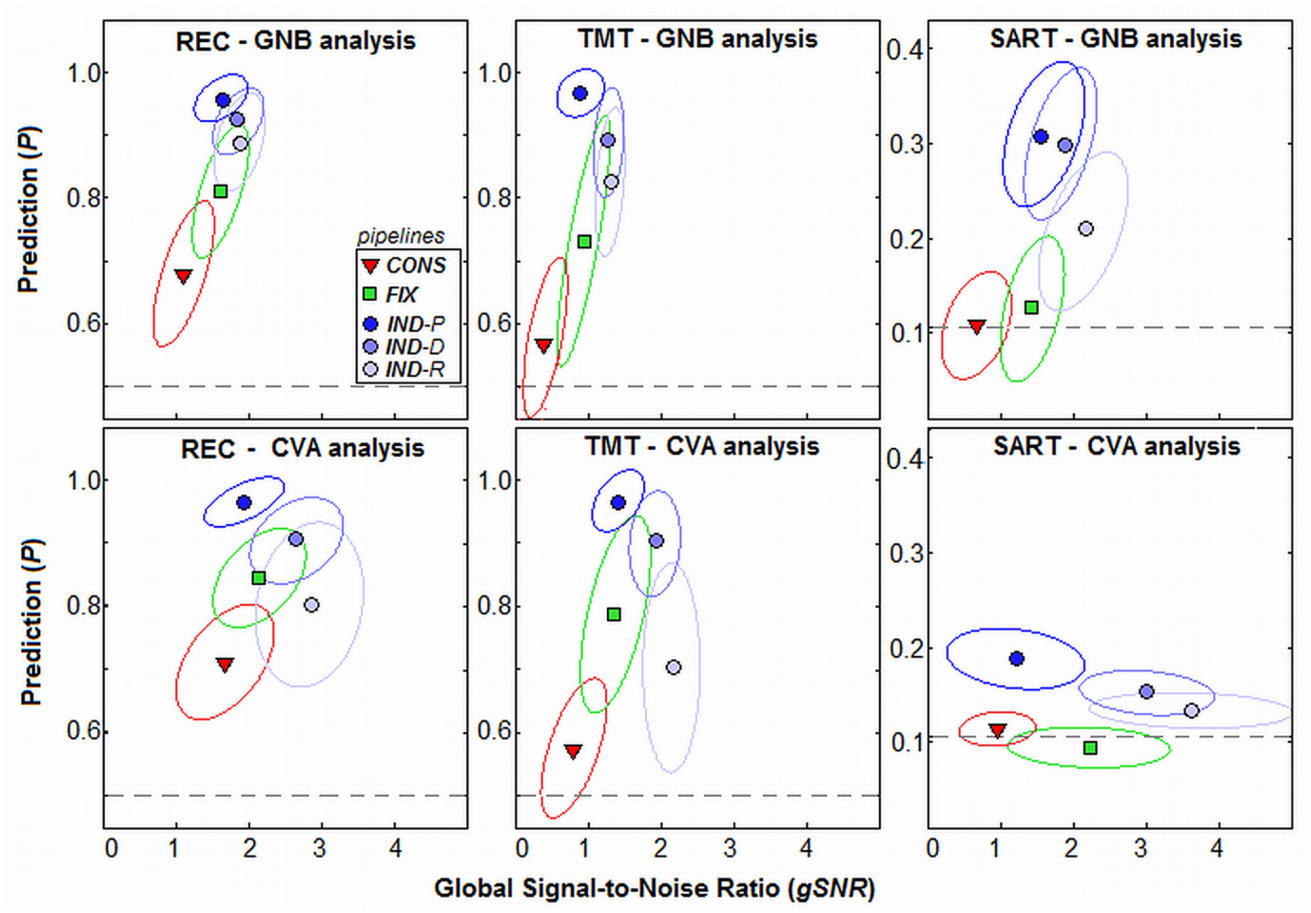
Prediction and global Signal-to-Noise Ratio for different preprocessing pipelines. Pipelines include a standard conservative pipeline (CONS), fixed optimization (FIX), and individual optimization maximizing prediction (IND-P), reproducibility (IND-R) or both metrics (IND-D). Each point shows average (gSNR, P) coordinates, for a different experimental task and analysis model, with ±1 Standard Deviation ellipses (enclosing ~68% of data points). Dashed lines indicate chance (random guessing) for prediction. Tasks include: Recognition (REC), Trail-Making Test (TMT) and Sustained Attention to Response Task (SART). Analysis models include: univariate Gaussian Naïve Bayes (GNB) and multivariate Canonical Variates Analysis (CVA). To see individual subject (gSNR, P) values, see S1 Fig.

Fig 3 plots metrics of pipeline performance, *gSNR* vs. *P*. The *gSNR* is a metric of signal detection that is monotonically related to spatial reproducibility *R* (refer to *METHODS*; *Optimization Metrics*). The mean (*P*, *gSNR*) values are plotted for the three tasks, and both GNB (top) and CVA (bottom) analysis models. Results are plotted for CONS, FIX and the three IND pipelines. For all tasks and analysis models, increasing model flexibility improves both *P* and *gSNR* metrics, where IND>FIX>CONS. The IND models also demonstrate a trade-off between metrics, as IND-P optimization produces (higher *P*, lower *gSNR*), IND-R produces (lower *P*, higher *gSNR*), and IND-D is intermediate between these two extremes. See S1 Fig for a plot of individual subject (*P*, *gSNR*) values.

**Fig 4:**
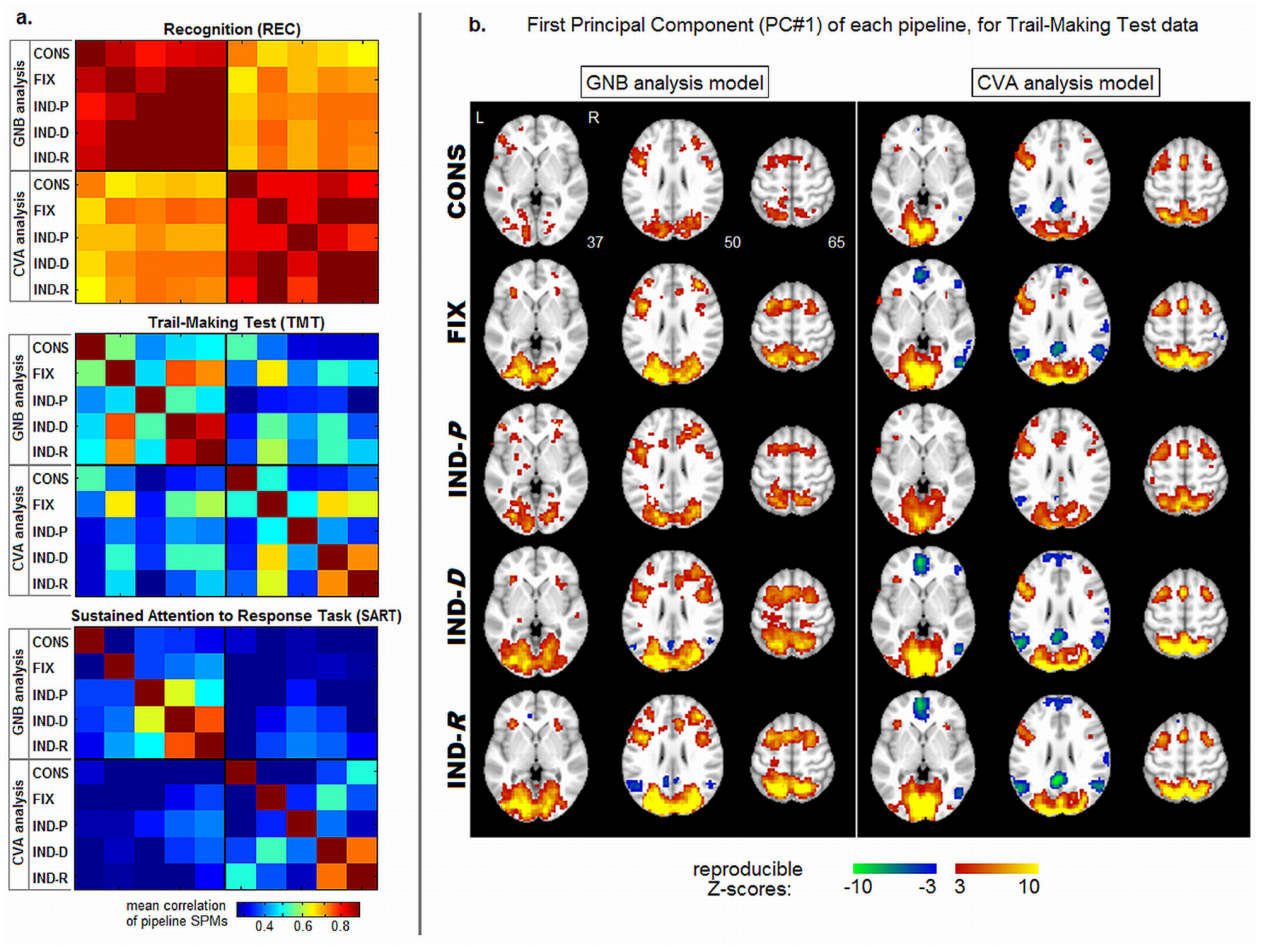
Spatial brain pattern similarity between preprocessing pipelines. (a) average correlation between pipeline SPMs, for Recognition (REC), Trail-Making Test (TMT) and Sustained Attention to Response Task (SART). Pipelines include: conservative (CONS) and optimal fixed (FIX), along with individually optimized pipelines based on prediction (IND-P), reproducibility (IND-R), and both metrics (IND-D); results are shown for univariate GNB analysis and multivariate CVA analysis. (b) the first Principal Component of subject SPMs for a representative TMT task, with GNB and CVA across five different preprocessing pipelines; these are the most stable spatial patterns across subjects. SPMs are Z-scored using a cross-validation procedure (*Optimization Metrics*), and thresholded at False-Discovery Rate FDR = .05 to correct for multiple comparisons.

The (*P*, *gSNR*) metrics reflect the quality of preprocessed data, but they provide no information about the similarity of the underlying spatial patterns of brain activation between pipelines. This is a critical issue, as neuroscience studies are often concerned with localizing the brain areas implicated in task performance. Therefore, we evaluated the relative similarity of SPM patterns for different pipeline optimization procedures. Fig 4a plots the average correlation between brain maps as a function of pipeline choice and analysis model, with results shown for the three different tasks. For the simple REC task, all pipeline brain maps have relatively high correlations, but mean correlations are comparatively low between CVA and GNB models. For the more complex TMT and event-related SART tasks, all correlations are decreased, and mean correlations between pipelines are more comparable to those between analysis models, although analysis model results are different regardless of the pipelines used. Therefore, pipeline choice has a greater impact on the spatial brain pattern for the more complex TMT task and event-related SART tasks, but the choice of analysis model is even more important.

For all tasks, the pipeline SPMZs with highest correlation are IND-R and IND-D, and FIX for all but SART where a more complicated similarity pattern is seen. This indicates that flexible pipelines optimized with *R* have the most consistent patterns, which tend to be quite similar to those for IND-D. Fig 4b demonstrates how pipeline choice alters the activation patterns. We plot the Z-scored first Principal Component in TMT data (i.e. the brain pattern of greatest variance across subjects’ SPMs), for each pipeline and analysis model. In these plots, Z-scores reflect the magnitude of reproducible activation across subjects (details in *Optimization Metrics*). In general, multivariate CVA detects sparser task-positive activations and greater task-negative activations, compared to univariate GNB. For both models, the CONS pipeline produces the most conservative extent of brain activations. FIX, IND-D and IND-R patterns are similar, with greater activation extent and magnitude, particularly in task-negative regions for CVA. The IND-P pipeline shows extensive task-positive signal, but weaker Z-score magnitudes. These results further demonstrate that flexible pipelines optimized with *R* have the most consistent patterns, with the greatest extent of reliable activations.

### Validation 1: Spatial Reliability of Independent Sessions

For the first validation measure, we assess the spatial reliability of activation patterns, between *independently-optimized* fMRI datasets. It is critical that fMRI activations have spatially reliable locations for repeated measures of a fixed task stimulus, in order to meaningfully interpret the brain regions recruited by a given task. We measure the overlap of active brain regions for (1) within-subject, between-session (test-retest run reliability, which is relevant to task learning studies and clinical assessments of disease progression and treatment), and (2) between-subject, within-session (which is relevant for group-level studies). We compared IND-D optimization against the standard CONS pipeline; IND-D is chosen as a representative pipeline, as it significantly improves both model prediction (P) and signal detection (gSNR) in all cases relative to CON and FIX (*p*<0.01, paired Wilcoxon tests), as shown in Fig 3. See S2 Fig for the effects of FIX optimization, and individual overlap values for both FIX and IND-D. The FIX results are omitted from the main text for clarity, as they are intermediate between CONS and IND; i.e. FIX significantly improves relative to CONS, and IND-D significantly improves relative to FIX.

**Fig 5:**
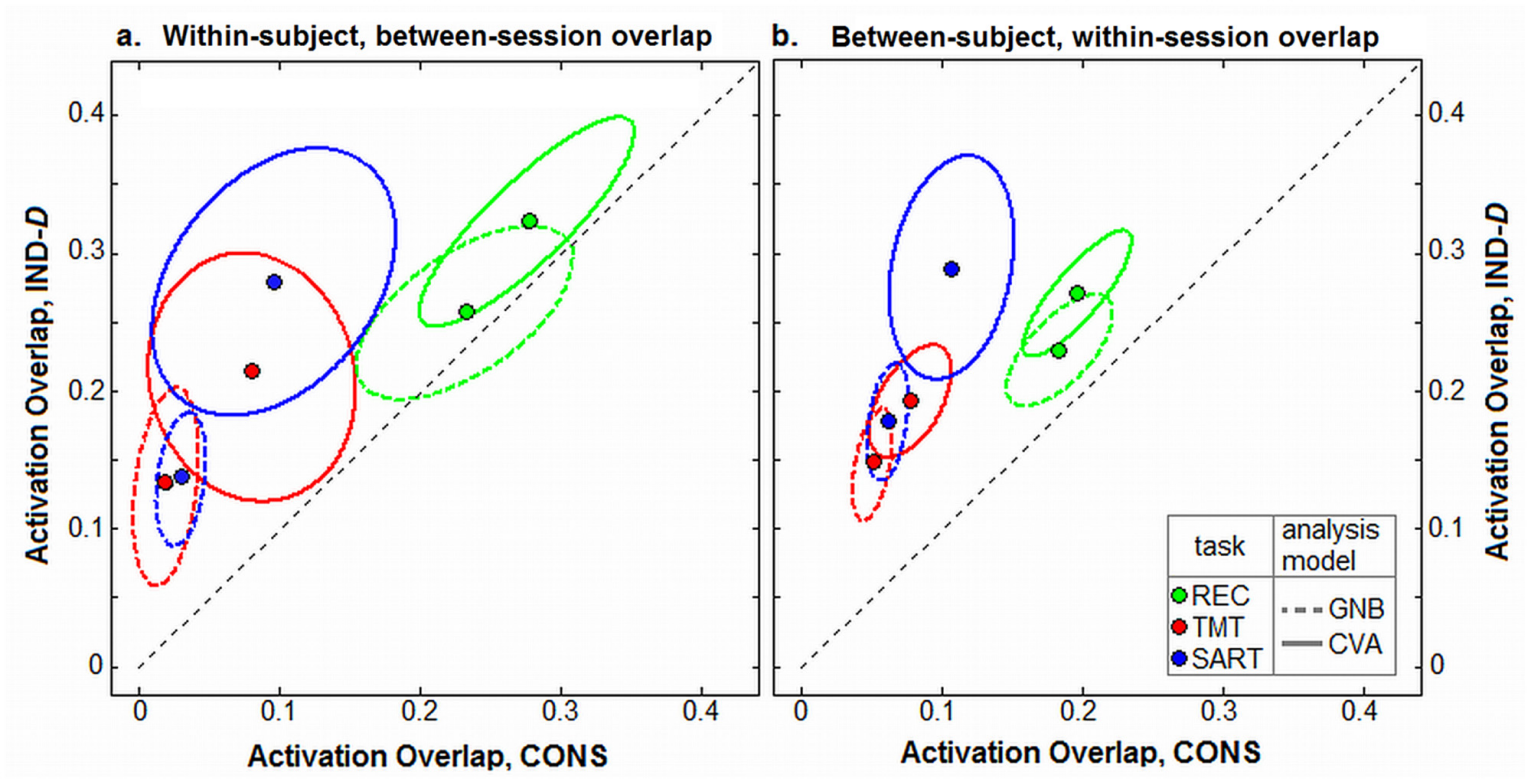
Change in activation overlap for individual subject optimization. Points represent average pairwise activation overlap between independently optimized pipelines, comparing standard conservative preprocessing (CONS) against individually optimized pipelines using the D(P, R) metric (IND-D). Results are shown for (a) within-subject between-session overlap, and (b) between-subject, within-session overlap. Overlap is measured by Jaccard index between SPMs at a False Discovery Rate = 0.05 threshold, for each task and analysis model. The ±1 Standard Deviation ellipses are also plotted (enclosing ~68% of data points). To see individual subject overlap values, see S2 Fig.

For each SPM, we identify significantly active brain voxels at a FDR = 0.05 threshold, to correct for multiple comparisons. We then measured the overlap of activated regions between pairs of SPMs, using the Jaccard index. Fig 5 compares the average overlap of brain maps for CONS vs. IND-D pipelines, for each of the 3 tasks and both GNB and CVA analysis models; the figure depicts the mean overlap across all subjects, along with ±1 the ± 1 Standard Deviation ellipse, enclosing ~68% of data points. The IND-D pipeline consistently improves average within-subject (Fig 5a) and between-subject (Fig 5b) activation overlap. All improvements are significant (paired Wilcoxon tests, *p*<0.001 for all). The relative improvement in overlap was greatest in more complex tasks. For example, mean between-subject overlap in REC increased by a factor of 1.5x, whereas for TMT it increased by 2.3x, and for SART, increased by a factor of 3.0x. See S2 Fig for the plot of individual subject pairwise overlap values.

### Validation 2: Estimating Brain-Behaviour Correlations

For the second validation, we measured the gSNR and predictive validity of behavioural correlations, for the different pipeline datasets. One of the main goals of fMRI is to link functional brain measures with behaviour. We therefore tested whether IND pipelines improve brain-behaviour correlations relative to CONS. To measure the amount of behavioural information captured in subjects’ SPMs for different pipelines, we performed a PLS analysis of SPMs against behavioural measures [64,65]. Using this model, we obtained: a Z-scored SPM that is correlated with behaviour, *gSNR*_behav_ of the pattern, and unbiased correlation of brain-pattern expression with behaviour (ρ_behav_). As in the previous section, results are shown for both CONS and IND-D pipelines, for each task and analysis model. See S3 Fig for the effects of FIX optimization; they tend to be better than CONS but comparable or lower in performance compared to IND-D.

**Fig 6:**
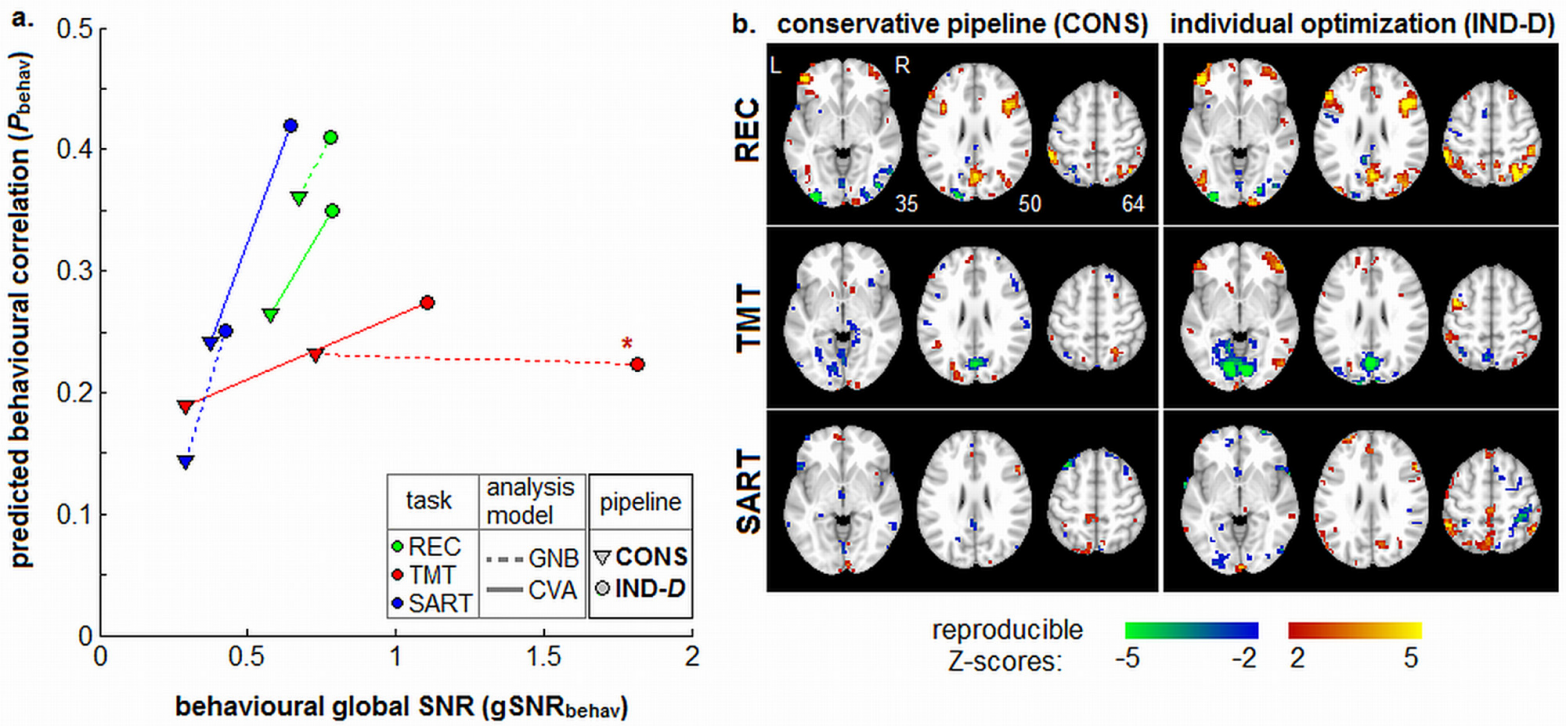
Change in behavioural correlation and global Signal to Noise Ratio for individual subject optimization. **(a)** global Signal-to-Noise Ratio (gSNR_behav_) vs. behavioural correlations (ρ_behav_), for Partial Least Squares (PLS) analysis of the correlation between SPM activation and behavioural performance. Results are shown for three tasks: Recognition (REC), Trail-Making Test (TMT) and Sustained Attention to Response Task (SART). We also plot results for two analysis models: univariate GNB and multivariate CVA. For each task/analysis model, we plot a line connecting (gSNR_behav_, ρ_behav_) from the standard conservative pipeline (CONS) to the individually optimized one (IND-D). IND-D optimization significantly improves gSNR_behav_ in all cases (p < 0.01), and significantly improves ρ_behav_ in all cases except TMT+GNB (marked with a ‘*’) at p ≤ 0.03 (see *METHDODS*: *Validation 2*: *Behavioural Testing*). **(b)** Z-scored SPMs showing brain regions that are most correlated with behavioural performance in PLS analysis, for all tasks and both pipelines, for the CVA model.

Fig 6a plots the median (ρ_behav_, *gSNR*_behav,_) values for PLS analysis of every task, and both GNB and CVA analysis models. We plot CONS vs. IND-D pipeline results, which are connected by a line for each task and analysis model. For all tasks and analysis models, the median *gSNR*_behav_ is significantly improved (*p*<0.01, Bootstrap significance estimates); median ρ_behav_ is significantly improved in all cases (*p* ≤ 0.03) except for TMT+GNB, where there is a non-significant change (*p* = 0.61). Fig 6b plots the Z-scored map of brain regions with the greatest behavioural correlations, for each task and pipeline for the CVA model. We observe activations that are consistent between CONS and IND-D pipelines, but IND-D produces larger reproducible Z-scores and more and larger activation regions for all tasks.

## Discussion

Our results demonstrate that a conservative preprocessing pipeline may strongly limit signal detection in fMRI, although depending on the task it may still achieve significant spatial pattern reliability and behavioural prediction. If pipeline steps are carefully chosen to optimize prediction and reproducibility, we can significantly improve independent tests of both the reliability of activation patterns within- and between-subjects, and correlations with independent behavioural measures. For this pipeline framework, we chose an extensive set of preprocessing choices, based on widespread literature use or previous pipeline testing [13–14,20–22,32]. However, the list of preprocessing steps is not meant to be definitive; our primary goal is to demonstrate the validity of the testing framework. A key feature of this framework is that it can incorporate novel preprocessing steps, and compare them against pre-existing pipeline options.

One major goal of developing this pipeline optimization framework is to reduce issues of subjective pipeline selection, which may produce highly biased, circular results. Common approaches of iteratively examining data and then applying preprocessing steps to correct for perceived noise/artifact can accidentally create results that are biased towards detecting some “expected” signal. And perhaps more importantly, the criteria used to select pipelines cannot be replicated in other studies. In the proposed framework, users are provided with pipelines that optimize the quantitative criterion of Euclidean distance D (*P*, *R*). The development of automated pipeline frameworks in fMRI may also help rectify issues of (a) inconsistent preprocessing descriptions, and (b) space limitations when reporting preprocessing methodology in fMRI literature. Under a standardized pipeline, the full sequence of preprocessing algorithms (and versions) can be reported individually, and users simply cite software, with a brief summary of chosen pipeline steps, e.g. in the form of S1 and S2 Tables, which avoids having to devote space to a full description of preprocessing steps.

### Summary of Findings

As shown in Fig 3, the choice of preprocessing pipeline determines the Prediction and *gSNR* (or reproducibility) of results. Constrained CONS and FIX pipelines produce lower *P* and *gSNR*, indicating relatively large model bias and variance, respectively [32,56]. In contrast, flexible IND pipelines produce significantly higher (*P*, *gSNR*). There is a trade-off in performance, depending on optimization criterion: IND-P has lower *gSNR*, whereas IND-R has lower *P*. This effect has been previously observed for multivariate classifiers, where weak regularization produces (higher *P*, lower *R*), and stronger regularization produces (lower *P*, higher *R*) [13,15,56]. Therefore, adaptive pipeline optimization serves a similar role as multivariate regularization, by constraining analysis models.

Fig 4 indicates that the greatest effect of pipelines on brain patterns depends on whether we perform flexible pipeline optimization using reproducibility. For univariate GNB, this primarily increases sensitivity to task-positive regions; multivariate CVA shows increased sensitivity to task-negative regions consistent with the Default-Mode [66,67], which is associated with spontaneous thought and decreased activity during external tasks. This is expected, as the DMN produces coherent BOLD fluctuations with high spatial reproducibility but weak correlations with task response. Thus, the GNB model (which is insensitive to inter-voxel correlations) and prediction optimization cannot reliably detect this network. Finally, we note that the CONS pipeline is least sensitive to both task-positive and -negative networks.

Pipeline optimization improves the overlap of independently-optimized SPMs (Fig 5). These results demonstrate that, even for relatively complex tasks and short task runs, we significantly improve test-retest and group reliability. This is particularly relevant in cases where it is critical to obtain reliable measures and where noise effects predominate, including clinical studies, and studies of aging and child populations [1–4]. We also demonstrate significant improvements in validity of behavioural studies (Fig 6), including better predicted behavioural correlations and greater stability of associated spatial patterns. These validation results strongly suggest that our optimized pipelines increase the sensitivity to brain regions that are reliably associated with behavioural performance, and are therefore likely to be associated with task-linked neuronal signals.

### Applications and Limitations

The task data were acquired during scanning sessions with multiple brief runs (<3 minutes each), for which we demonstrate the ability to detect strong, reliable activations that are comparable to standard experimental datasets. The brief design is also relevant for clinical applications, in which scanning must be brief to ensure patient compliance, and large portions of data may be discarded due to poor behavioural performance or artifact [1,3]. It is not yet known whether the benefit of pipeline optimization is comparable for standard experimental datasets with far more data points. It is possible that the power increase reduces the impact of pipeline choice. However, preliminary resting-state studies [68] indicate a comparable benefit of pipeline optimization across a range of different sample sizes. In addition, increased scanning time only guarantees increased power if BOLD effects are stationary. If more data is acquired in the presence of dynamic BOLD changes, this may increase the variance of signal estimates. This underscores the potential importance of improving signal quality in shorter epoch datasets, and the impact of epoch length should be further investigated in future research.

From an application standpoint, adaptive pipeline optimization raises potential concerns of comparability between different groups. But it is not always appreciated that adaptive preprocessing is already in widespread use for fMRI, particularly when comparing groups that differ systematically in signal/noise parameters. Component models based on ICA are routinely fit to data from individual subjects, leading to different orders of regression model per subject [69]. Similarly, “scrubbing” protocols that discard or interpolate scan volumes per subject alter the temporal smoothness of data in different ways, depending on the number of discarded outliers [42,43]. While such practises are well-established in the fMRI literature, it does not guarantee that they are appropriate preprocessing strategies, and adaptive methods may lead to spurious results that are driven by processing algorithms rather than brain function. In general, highly flexible preprocessing techniques should be examined with caution and carefully validated using multiple different datasets and metrics to assess for potential biases.

The principal issue is whether adaptive preprocessing increases model over-fit relative to a fixed model. As demonstrated in this paper, IND optimization significantly improves generalization, based on multiple validation metrics. Prior simulation studies have also established that IND is not significantly more biased than FIX pipelines [22], and we have previously shown that the advantageous or deleterious impact of particular choices (e.g. motion parameter regression) may depend on the magnitude of structured signal artefacts (e.g. motion amplitude) [21]. That being said, no model is completely free from bias, and all preprocessing choices may have systematic effects on results. For this reason, we would advocate the reporting of preprocessing choices, and post-hoc examination of IND and FIX results, to gain a better understanding of pipeline effects, along with the reporting of independent performance measures such as between-subject activation overlap and levels of behavioural prediction. In addition, we would emphasize that it is critical to test all preprocessing steps being applied to one’s fMRI data within this optimization framework. Otherwise, the “pre-selection” of a subset of pipeline steps may re-introduce user-dependent biases into the pipeline optimization framework.

A major advantage of the proposed cross-validation model is that it does not require correction for degrees of freedom (*dof*) when comparing pipelines. In standard significance testing, model fit can be trivially inflated by increasing *dof*, but an overly-flexible model will exhibit poor reproducibility (*R*) and predictive generalization (*P*). Thus, the optimization framework avoids cases where it is more challenging to compare *dof* between models, e.g. in standard null-hypothesis testing and theoretic estimators of model fit, such as Bayes Information Criterion (BIC) and Akaike Information Criterion (AIC). For example, while *dof* can be easily estimated for GLM regressors, it is less clear how spatial smoothing and outlier censoring constrains the data space and subsequent analyses. Since the split-half model obtains cross-validated estimates of signal and noise, they can be directly used for second level group analyses, such as the PLS behavioural analyses performed in this paper. Similarly, other post-hoc analyses on the optimally preprocessed data can be used in a cross-validation framework, or for measuring reliability of activations. Conversely, single-subject inferential testing based on p-values would have to keep track of the regression models used for individual subjects.

The assumptions of our pipeline framework must also be considered in the context of the data that is being optimized. Our framework identifies the pipeline with the most reproducible SPM and optimal prediction between within-run data splits. In cases where we expect changes in the patterns of brain activity over the course of a task run (e.g. a motor learning task), this is inappropriate, as the model will treat these changes as non-reproducible confounds. However, as we show, a sequence of short runs may be individually optimized, which is suitable for probing learning changes over multiple practice sessions. As a rule, optimization should be done at the largest time-scale on which the hypothesis presumes stationary BOLD signal. This paper shows such an optimization approach in practise, as we adaptively fit different pipelines for run 1 and run 2 within a single scanning session, across multiple tasks.

### Future Research

In this paper we focused principally on the effects of including/excluding a fixed order of pipeline steps. One may also consider permuting the order in which preprocessing pipelines are applied. This has only been examined in limited contexts, partly due to the combinatorial explosion of possible ways in which pipeline steps may be ordered. For example, the relative order of motion correction, RETROICOR and slice-timing has been investigated [28]; the order in which spectral filtering, de-spiking and nuisance regression is performed has also been tested [29]. In some cases there is a well-motivated ordering of steps, e.g. spectral filtering should not precede nuisance regression, as it will introduce artifactual frequencies. In other cases, it is less clear. For example, should de-spiking precede nuisance regression, or be performed afterwards? This is an area that must be investigated in future work.

Another area of future investigation lies in the optimization of spatial preprocessing methods such as the choice of smoothing kernels. It is well established that some fixed spatial smoothing is beneficial at the group level, and that it significantly improves various performance metrics [15][70]. But while studies have consistently demonstrated the benefit of individual subject optimization for temporal processing methods (e.g. regression of nuisance covariates), less is known about individual spatial processing optimization. According to the matched filter theorem [71], the choice of smoothing scale dictates the size of detected activations, and so it may be desirable to employ a “2-stage” optimization process, wherein the optimal fixed smoothing kernel is chosen across subjects, and temporal processing steps are optimized at the individual subject level. However, there is some evidence that smoothing can also be successfully optimized at the individual level [14], and some studies even advocate adapting smoothing as a function of both dataset and brain region [72].

Another, more challenging issue is the integration of Quality Control (QC) protocols into pipeline optimization. QC measures serve a distinct but complementary role relative to pipeline optimization, in identifying the regularities of data, and identifying datasets where the pre-existing preprocessing tools cannot adequately correct for artifact. In many ways, it is a more challenging prospect to develop a comprehensive QC protocol, as it requires that one catalogue all of the ways in which artifacts can occur, whereas pipeline optimization is primarily driven by a fixed set of optimization metrics. While this is a recognized issue, there are currently no agreed-upon guidelines for fMRI QC [23, 25], although suggested protocols have been developed [73] and packages have been developed for public use (e.g. www.nitrc.org/projects/artifact_detect). This remains an area of active research, and may require multiple stages. That is, QC prior to preprocessing optimization and afterwards, in order to verify that pipeline optimization was successful.

## Supporting Information

S1 Fig Prediction and global Signal-to-Noise Ratio for different preprocessing pipelines. Pipelines include a standard conservative pipeline (CONS), fixed optimization (FIX), and individual optimization maximizing prediction (IND-P), reproducibility (IND-R) or both metrics (IND-D). Large icons show average (gSNR, P) coordinates, for a different experimental task and analysis model, with ±1 Standard Deviation ellipses (enclosing ~68% of data points). Dashed lines indicate chance (random guessing) for prediction. Scatter points represent individual subject (gSNR, P) values. Tasks include: Recognition (REC), Trail-Making Test (TMT) and Sustained Attention to Response Task (SART). Analysis models include: univariate Gaussian Naïve Bayes (GNB) and multivariate Canonical Variates Analysis (CVA).
(PNG)

S2 Fig Change in activation overlap for optimal fixed and individual subject pipelines. Large icons represent average pairwise activation overlap between independently optimized pipelines, comparing standard conservative preprocessing (CONS) against the optimal fixed pipeline (FIX) and individually optimized pipelines (IND-D), both optimized using the D(P, R) metric. Scatter points represent individual subject overlap values. Results are shown for **A**. within-subject between-session overlap, and **B**. between-subject, within-session overlap. Overlap is measured by Jaccard index between SPMs at a False Discovery Rate = 0.05 threshold, for each task and analysis model. The ±1 Standard Deviation ellipses are also plotted (enclosing ~68% of data points).
(PNG)

S3 Fig Change in behavioural correlation and global Signal to Noise Ratio for optimal fixed and individual subject pipelines. We plot global Signal-to-Noise Ratio (gSNR_behav_) vs. behavioural correlations (ρ_behav_), for Partial Least Squares (PLS) analysis of the correlation between SPM activation and behavioural performance. Results are shown for three tasks: Recognition (REC), Trail-Making Test (TMT) and Sustained Attention to Response Task (SART). We also plot results for two analysis models: univariate GNB and multivariate CVA. For each task/analysis model, we plot a line connecting (gSNR_behav_, ρ_behav_) from the standard conservative pipeline (CONS) to (a) the optimal fixed pipeline (FIX), and (b) the individually optimized pipeline (IND-D). FIX and IND-D data-points are represented by circles; CONS data-points are represented by triangles.
(PNG)

S1 Table Fraction of subjects that include each preprocessing step under different pipeline optimization approaches, for univariate Gaussian Naïve Bayes. Darker shading indicates a greater fraction of subjects. Results are shown for tasks: Recognition (REC), Trail-Making Test (TMT) and Sustained Attention to Response Task (SART). Pipelines include conservative preprocessing (CONS), fixed optimal pipelines (FIX), and individual pipelines optimized with prediction (IND-P), reproducibility (IND-R) and both metrics (IND-D). Preprocessing steps include: motion correction (MC), censoring outliers (CENS), physiological correction with RETROICOR (RET), slice-timing correction (STC), motion parameter regression (MPR), including task design regressor (TASK), global signal regression (GSPC1), physiological correction with PHYCAA+ (PHY+) and temporal detrending (DET). For DET, we plot the median [minimum, maximum] detrending order for each task and pipeline.
(DOCX)

S2 Table Fraction of subjects that include each preprocessing step under different pipeline optimization approaches, for multivariate Canonical Variates Analysis. Darker shading indicates a greater fraction of subjects. Results are shown for tasks: Recognition (REC), Trail-Making Test (TMT) and Sustained Attention to Response Task (SART). Pipelines include conservative preprocessing (CONS), fixed optimal pipelines (FIX), and individual pipelines optimized with prediction (IND-P), reproducibility (IND-R) and both metrics (IND-D). Preprocessing steps include: motion correction (MC), censoring outliers (CENS), physiological correction with RETROICOR (RET), slice-timing correction (STC), motion parameter regression (MPR), including task design regressor (TASK), global signal regression (GSPC1), physiological correction with PHYCAA+ (PHY+) and temporal detrending (DET). For DET, we plot the median [minimum, maximum] detrending order for each task and pipeline.
(DOCX)

S3 Table Summary of significant outlier datasets, for different tasks and pipelines. We list significant outliers in behavioural metrics, and in fMRI data (see Supplementary Note 4 for fMRI outlier testing procedure). We also list the number of remaining runs, out of the original 94 runs.
(DOCX)

S1 Text Censoring Outlier Brain Volumes. This supporting text provides a detailed description of the algorithm used to detect “spikes” created by abrupt head motion during scanning, which are then removed by interpolating neighbouring voxel values.
(DOCX)

S2 Text Finding Pipelines with Task-Coupled Head Motion. In this text, we define the algorithm that is used to identify subject pipelines that produce activation maps with significant motion artifact, identified via significant weighting of brain edges.
(DOCX)

S3 Text Reproducible Principal Component Brain Maps. This procedure is used to obtain reproducible Z-scored brain patterns that explain the greatest variance within a set of subject activation maps.
(DOCX)

S4 Text Split-half Behavioural Partial Least Squares. This algorithm is used to identify brain patterns that have greatest covariance with behaviour, within a set of subject activation maps. It is estimated in a split-half cross-validation framework in order to obtain reproducible Z-scored brain patterns and unbiased measures of behavioural correlation.
(DOCX)

S5 Text Identifying Outlier Subjects before Group Analysis. This procedure is used to detect subjects with activation patterns that are significant outliers, based on their influence in multivariate Principal Component space. This allows us to remove them prior to behavioural analyses, in order to improve the stability of results.
(DOCX)
