# Correction: An Automated, Adaptive Framework for Optimizing Preprocessing Pipelines in Task-Based Functional MRI

**DOI:** 10.1371/journal.pone.0145594

**Published:** 2015-12-17

**Authors:** Nathan W. Churchill, Robyn Spring, Babak Afshin-Pour, Fan Dong, Stephen C. Strother

There is an error in Fig 6. Please view the correct Fig 6 here.

**Fig 6:**
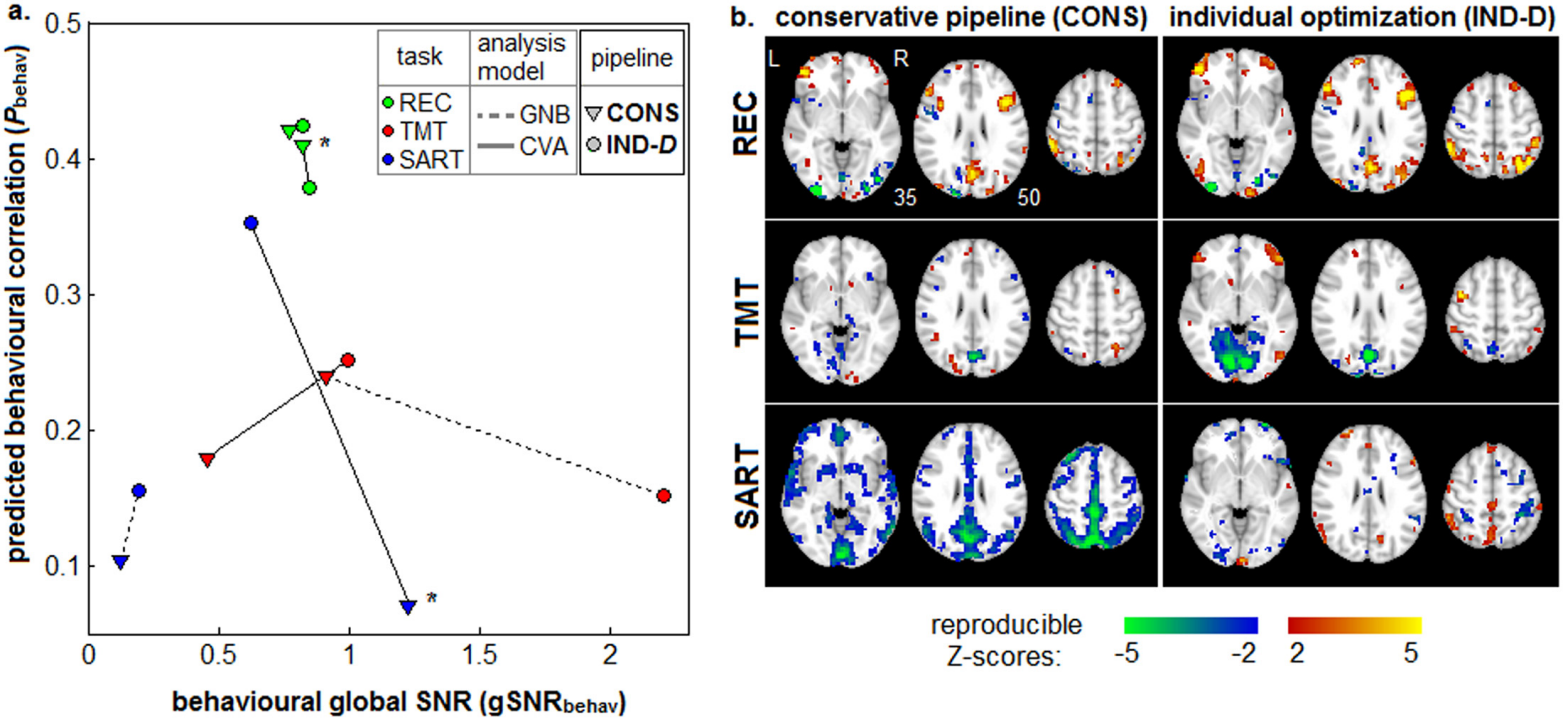
Change in behavioural correlation and global Signal to Noise Ratio for individual subject optimization. (a) global Signal-to-Noise Ratio (gSNR_behav_) vs. behavioural correlations (ρ_behav_), for Partial Least Squares (PLS) analysis of the correlation between SPM activation and behavioural performance. Results are shown for three tasks: Recognition (REC), Trail-Making Test (TMT) and Sustained Attention to Response Task (SART). We also plot results for two analysis models: univariate GNB and multivariate CVA. For each task/analysis model, we plot a line connecting (gSNR_behav_, ρ_behav_) from the standard conservative pipeline (CONS) to the individually optimized one (IND-D). IND-D optimization significantly improves gSNR_behav_ in all cases (p < 0.01), and significantly improves ρ_behav_ in all cases except TMT+GNB (marked with a ‘*’) at p ≤ 0.03 (see *METHDODS*: *Validation 2*: *Behavioural Testing*). **(b)** Z-scored SPMs showing brain regions that are most correlated with behavioural performance in PLS analysis, for all tasks and both pipelines, for the CVA model.

There is an error in the second paragraph of the Results section under the subheading Validation 2: Estimating Brain-Behaviour Correlations. The correct paragraph should read: Fig 6a plots the median (ρ_behav_, *gSNR*_behav_,) values for PLS analysis of every task, and both GNB and CVA analysis models. In general, IND optimization improves model performance. It significantly improves ρ_behav_ for all models (p<0.001, bootstrapped significance) except TMT+GNB (significant at p<0.001) and REC+CVA(non-significant at p = 0.28). It also significantly improves *gSNR*_*behav*_ for all models except SART+CVA (marginally worse at p = 0.03). Fig 6b plots Z-scored maps of brain regions with the greatest behavioural correlations, for each task and pipeline of the CVA model. For REC and TMT, we observe similar activation patterns between CONS and IND-D pipelines, although IND-D produces higher reproducible Z-scores and more extensive activation regions for all tasks. Whereas for SART, CONS produces more extensive activation than IND-D, albeit with limited spatial specificity.
