# Supplementary figures and images for: An Automated, Adaptive Framework for Optimizing Preprocessing Pipelines in Task-Based Functional MRI
Source: PLoS One. 2015 Jul 10;10(7):e0131520. doi: 10.1371/journal.pone.0131520 (PMC4498698; doi:10.1371/journal.pone.0131520)

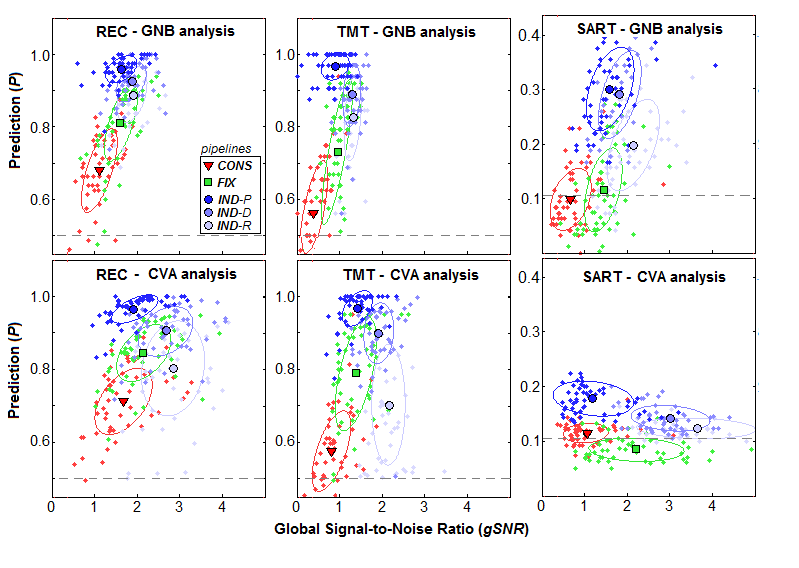

Supplement: S1 Fig — Pipelines include a standard conservative pipeline (CONS), fixed optimization (FIX), and individual optimization maximizing prediction (IND-P), reproducibility (IND-R) or both metrics (IND-D). Large icons show average (gSNR, P) coordinates, for a different experimental task and analysis model, with ±1 Standard Deviation ellipses (enclosing ~68% of data points). Dashed lines indicate chance (random guessing) for prediction. Scatter points represent individual subject (gSNR, P) values. Tasks include: Recognition (REC), Trail-Making Test (TMT) and Sustained Attention to Response Task (SART). Analysis models include: univariate Gaussian Naïve Bayes (GNB) and multivariate Canonical Variates Analysis (CVA). (PNG) [file pone.0131520.s001.png]

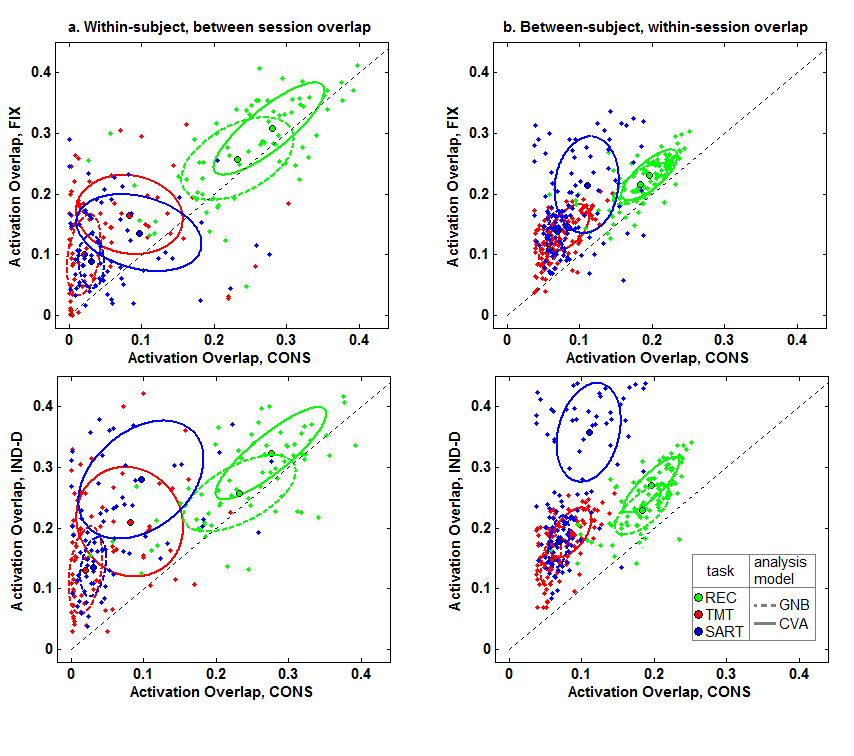

Supplement: S2 Fig — Large icons represent average pairwise activation overlap between independently optimized pipelines, comparing standard conservative preprocessing (CONS) against the optimal fixed pipeline (FIX) and individually optimized pipelines (IND-D), both optimized using the D(P, R) metric. Scatter points represent individual subject overlap values. Results are shown for A. within-subject between-session overlap, and B. between-subject, within-session overlap. Overlap is measured by Jaccard index between SPMs at a False Discovery Rate = 0.05 threshold, for each task and analysis model. The ±1 Standard Deviation ellipses are also plotted (enclosing ~68% of data points). (PNG) [file pone.0131520.s002.png]

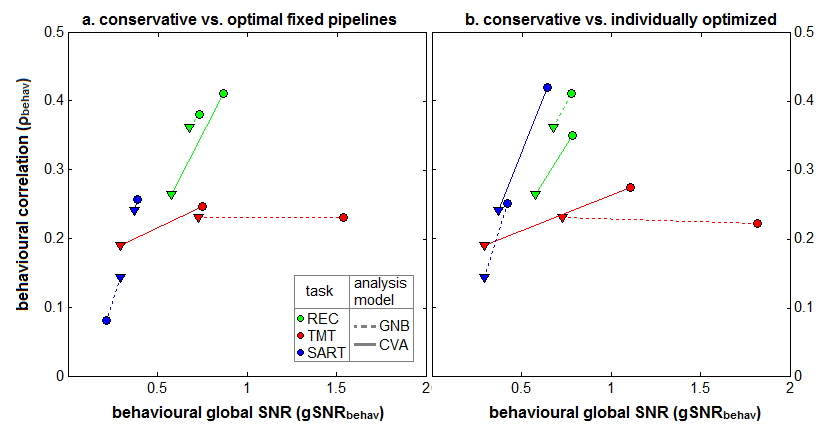

Supplement: S3 Fig — We plot global Signal-to-Noise Ratio (gSNRbehav) vs. behavioural correlations (ρbehav), for Partial Least Squares (PLS) analysis of the correlation between SPM activation and behavioural performance. Results are shown for three tasks: Recognition (REC), Trail-Making Test (TMT) and Sustained Attention to Response Task (SART). We also plot results for two analysis models: univariate GNB and multivariate CVA. For each task/analysis model, we plot a line connecting (gSNRbehav, ρbehav) from the standard conservative pipeline (CONS) to (a) the optimal fixed pipeline (FIX), and (b) the individually optimized pipeline (IND-D). FIX and IND-D data-points are represented by circles; CONS data-points are represented by triangles. (PNG) [file pone.0131520.s003.png]
