# Supplementary material for: An Automated, Adaptive Framework for Optimizing Preprocessing Pipelines in Task-Based Functional MRI
Source: PLoS One. 2015 Jul 10;10(7):e0131520. doi: 10.1371/journal.pone.0131520 (PMC4498698; doi:10.1371/journal.pone.0131520)

**Table S2**: **fraction of subjects that include each preprocessing step under different pipeline optimization approaches, for multivariate Canonical Variates Analysis.** Darker shading indicates a greater fraction of subjects. Results are shown for tasks: Recognition (REC), Trail-Making Test (TMT) and Sustained Attention to Response Task (SART). Pipelines include conservative preprocessing (CONS), fixed optimal pipelines (FIX), and individual pipelines optimized with prediction (IND-P), reproducibility (IND-R) and both metrics (IND-D). Preprocessing steps include: motion correction (MC), censoring outliers (CENS), physiological correction with RETROICOR (RET), slice-timing correction (STC), motion parameter regression (MPR), including task design regressor (TASK), global signal regression (GSPC1), physiological correction with PHYCAA+ (PHY+) and temporal detrending (DET). For DET, we plot the median [minimum, maximum] detrending order for each task and pipeline.


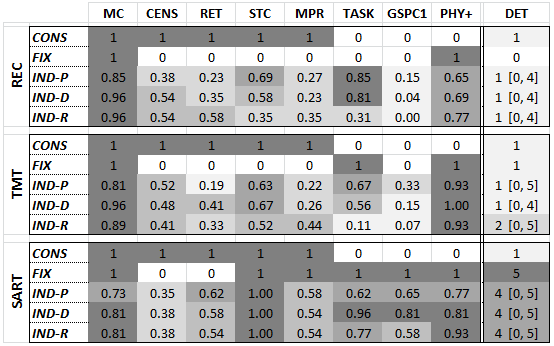

Supplement: S2 Table — Darker shading indicates a greater fraction of subjects. Results are shown for tasks: Recognition (REC), Trail-Making Test (TMT) and Sustained Attention to Response Task (SART). Pipelines include conservative preprocessing (CONS), fixed optimal pipelines (FIX), and individual pipelines optimized with prediction (IND-P), reproducibility (IND-R) and both metrics (IND-D). Preprocessing steps include: motion correction (MC), censoring outliers (CENS), physiological correction with RETROICOR (RET), slice-timing correction (STC), motion parameter regression (MPR), including task design regressor (TASK), global signal regression (GSPC1), physiological correction with PHYCAA+ (PHY+) and temporal detrending (DET). For DET, we plot the median [minimum, maximum] detrending order for each task and pipeline. (DOCX) [file pone.0131520.s005.docx]
