# Supplementary material for: An Automated, Adaptive Framework for Optimizing Preprocessing Pipelines in Task-Based Functional MRI
Source: PLoS One. 2015 Jul 10;10(7):e0131520. doi: 10.1371/journal.pone.0131520 (PMC4498698; doi:10.1371/journal.pone.0131520)
Supplement: S3 Table — We list significant outliers in behavioural metrics, and in fMRI data (see Supplementary Note 4 for fMRI outlier testing procedure). We also list the number of remaining runs, out of the original 94 runs. (DOCX) [file pone.0131520.s006.docx]

**Table S3**: **summary of significant outlier datasets, for different tasks and pipelines**. We list significant outliers in behavioural metrics, and in fMRI data (see Supplementary Note 4 for fMRI outlier testing procedure). We also list the number of remaining runs, out of the original 94 runs.

|  | **behavioural outliers** | **fMRI outliers, CONS** | **fMRI outliers, IND-D** | **Remaining runs, CONS** | **Remaining runs, IND-D** |
| --- | --- | --- | --- | --- | --- |
| **REC** | 2 | 8 | 8 | 82 | 84 |
| **TMT** | 5 | 6 | 4 | 83 | 85 |
| **SART** | 4 | 5 | 7 | 85 | 83 |
