# Supplementary material for: An Automated, Adaptive Framework for Optimizing Preprocessing Pipelines in Task-Based Functional MRI
Source: PLoS One. 2015 Jul 10;10(7):e0131520. doi: 10.1371/journal.pone.0131520 (PMC4498698; doi:10.1371/journal.pone.0131520)
Supplement: S1 Text — This supporting text provides a detailed description of the algorithm used to detect “spikes” created by abrupt head motion during scanning, which are then removed by interpolating neighbouring voxel values. (DOCX) [file pone.0131520.s007.docx]

**Text S1:** Censoring Outlier Brain Volumes

This section defines a testing procedure, first applied by Campbell et al.**^45^**, that identifies outliers in fMRI data that are potentially caused by abrupt head motion. The algorithm identifies and removes timepoints that are outliers in both the 6 rigid-body motion parameter estimates (MPEs), and in the fMRI data after performing motion correction. For an fMRI data matrix ***X***_fmri_ (with dimensions *V* voxels x *T* timepoints) with a matrix of MPE time-courses ***X***_mpe_ (6 x *T*), a robust procedure for removing spikes is defined as follows:

1. decompose ***X***_fmri_ and ***X***_mpe_ using PCA, and represent the data in PC space coordinates, as ***Q***_fmri_ (with dimensions *T* x *T*) and ***Q***_mpe_ (6 x *T*). This provides an orthonormal basis that maximizes the explained variance in the data, and greatly reduces the dimensionality of fMRI data.
2. For PC-space data-points ***q***_t_ (1 < *t* < *T*), obtain the median coordinate vector ***q***_med(t)_ within a 15-TR time window centered at *t* (e.g. all volumes within the ±7 TR of volume *t*). For volumes at the start (*t*<8) and end (*t*>*T*-7) of the run, this will be less than 15 time-points. Then compute the squared Euclidean distance $d_{t}=\left\| \boldsymbol{q}_{t}-\boldsymbol{q}_{med(t)} \right\|^{2}$. This measures the displacement of ***q***_t_ away from surrounding data points; a point ***q***_t_ with larger displacement *d*_t_ is more likely to be an outlier. This procedure is performed for all data points in ***Q***_fmri_ and ***Q***_mpe_, producing *T*x1 vectors of displacement values ***d***_fmri_ and ***d***_mpe_, corresponding to timepoints in the fMRI data.
3. For each ***d***, fit a Gamma probability distribution to the data, by computing the maximum likelihood estimates of the distribution parameters. The Gamma model is used, as it forms a flexible distribution over a set of random, strictly positive variables. Then identify timepoints that are outliers at *p*<0.05, for both ***d***_fmri_ and ***d***_mpe_ distributions. These are labeled as motion outliers in the data.
4. Remove any outlier fMRI volumes, and replace them by interpolating voxel values from adjacent volumes, using cubic splines. This controls for potential spikes, while minimizing discontinuities in the fMRI time-courses due to removal of outliers.

This provides an adaptive, statistically-driven procedure, used to remove spikes in fMRI data.
