# Supplementary material for: An Automated, Adaptive Framework for Optimizing Preprocessing Pipelines in Task-Based Functional MRI
Source: PLoS One. 2015 Jul 10;10(7):e0131520. doi: 10.1371/journal.pone.0131520 (PMC4498698; doi:10.1371/journal.pone.0131520)
Supplement: S2 Text — In this text, we define the algorithm that is used to identify subject pipelines that produce activation maps with significant motion artifact, identified via significant weighting of brain edges. (DOCX) [file pone.0131520.s008.docx]

**Text S2:** Finding Pipelines with Task-Coupled Head Motion

One of the challenges of optimization based on prediction accuracy and spatial reproducibility (*P*, *R*), is that head motion may also be correlated with stimuli, and induce signal change in spatially consistent brain regions, particularly brain edges where there is a rapid change in magnetic susceptibility. These effects are difficult to separate from BOLD signal, and may drive model optimization if BOLD signal has comparatively low (*P*, *R*). This section established a test, initially defined for Churchill et al.**^22^**, designed to detect when model optimization is driven by task-coupled motion (TCM) based on the spatial distribution of signal in the SPMs:

For a set of *S* subjects, identify the optimal pipeline with rigid-body motion correction (MOTCOR) fixed on for each subject. It is assumed that MOTCOR provides a minimum level of control against head motion; no pipeline choice that excludes MOTCOR should significantly increase the amount of motion artifact.

For each subject (1 < *s* < *S*):

1. Estimate edge artifact structure: Take the 4D time-series of the optimized pipeline and compute the first-order spatial derivative at each voxel (i.e. the difference of adjacent voxel values for each time-point). This derivative is taken separately along each of the (X,Y,Z) axes, generating three 4D datasets; each approximates signal change caused by head motion < 1 voxel along one axis, for every brain volume. PCA is performed on each time-series, and the first eigenimage retained, which typically accounts for >95% of total variance. This produces 3 derivative eigenimages, stored in matrix ***G*** (*V* voxels x 3).

2. Measure correlation with edge artifact: Perform spatial Canonical Correlations Analysis (CCA) between the optimal SPM (with MOTCOR) and ***G***. This is the multivariate extension of Pearson correlation, which gives canonical correlation ρ_k,MOTCOR-ON_ between the SPM and the linear combination of ***G*** vectors that is most correlated with the SPM, i.e. the best linear approximation any motion artifact present in the SPM.

Given *S* data sets with MOTCOR applied and their artifact-correlation values {ρ_k, mc_}, remove any outliers greater than 3 standard deviations above the mean; this accounts for cases where MOTCOR entirely fails to control motion (at *p*=0.001), assuming this is relatively rare. Then, record the highest remaining value in {ρ_k,MOTCOR-ON_} for the current dataset. This value, denoted **ρ_max_**, is the empirical threshold of significant motion artifact.

Identify any subject’s data set that is optimized without MOTCOR. Estimate {ρ_k,MOTCOR-OFF_} as outlined above for each data set. If ρ_MOTCOR-OFF_ < **ρ_max_**, keep this pipeline without MC; otherwise, select the optimal pipeline with MOTCOR on. Perform the same testing and re-optimization for subjects with MC identified as outliers in (**2**).

This model provides a conservative test for TCM artifact that may be adapted to any dataset or set of pipelines.
