# Supplementary material for: An Automated, Adaptive Framework for Optimizing Preprocessing Pipelines in Task-Based Functional MRI
Source: PLoS One. 2015 Jul 10;10(7):e0131520. doi: 10.1371/journal.pone.0131520 (PMC4498698; doi:10.1371/journal.pone.0131520)
Supplement: S3 Text — This procedure is used to obtain reproducible Z-scored brain patterns that explain the greatest variance within a set of subject activation maps. (DOCX) [file pone.0131520.s009.docx]

**Text S3:** Reproducible Principal Component Brain Maps

Given a group of subject SPMs, a common goal is to summarize them by describing the most common spatial patterns expressed within the set of SPMs. This can be done by using Principal Component Analysis to produce spatial eigenimages, representing the greatest variance across SPMs. However, this lacks any information about the statistical reliability or importance of brain regions in the eigenimages. In this section, we define a procedure for generating reproducible Z-scored spatial eigenimages, using an adaptation of the NPAIRS split-half framework.

Given a set of *S* subject SPMs, we concatenate them into a data matrix ***X*** (of dimensions: *V* voxels x *S*). For 100 iterations:

1. Randomly split the data in half, producing matrices ***X***_1_, ***X***_2_, of dimensions (*V* x *S*/2)
2. Perform Singular Value Decomposition (SVD) ***X***_i_ = ***U***_i_***Λ***_i_***V***_i_^T^ (i=1,2) where ***U***_i_ is a set of orthonormal image basis vectors, ***Λ***_i_ is a diagonal matrix of singular values, and ***V***_i_ is a set of orthonormal subject-weight vectors. Retain the first spatial eigenimage ***u***_1(i)_, which accounts for the most data variance in the set of SPMs
3. Compute an rSPMZ using the split-half eigenimages ***u***_1(1)_ and ***u***_1(2)_, and the procedure defined in the *Optimization Metrics* section of METHODS.

We then measure the average rSPMZ across all resampling splits. This provides a Z-scored estimate of the stable brain pattern that expresses the greatest variance across subjects, where higher Z-scores reflect increased signal reproducibility.
