# Supplementary material for: An Automated, Adaptive Framework for Optimizing Preprocessing Pipelines in Task-Based Functional MRI
Source: PLoS One. 2015 Jul 10;10(7):e0131520. doi: 10.1371/journal.pone.0131520 (PMC4498698; doi:10.1371/journal.pone.0131520)
Supplement: S4 Text — This algorithm is used to identify brain patterns that have greatest covariance with behaviour, within a set of subject activation maps. It is estimated in a split-half cross-validation framework in order to obtain reproducible Z-scored brain patterns and unbiased measures of behavioural correlation. (DOCX) [file pone.0131520.s010.docx]

**Text S4:** Split-half Behavioural Partial Least Squares

This procedure was first established in Churchill et al.[**30**]. For each of *S* subjects, we have a single SPM consisting of *V* voxels, concatenated into (*V* x *S*) data matrix ***X***; we also have a single behavioural measure for each subject, concatenated into an (S x 1) behaviour vector ***b***. For 100 resampling splits:

- - - 1. Randomly split the data in half, producing matrices ***X***_1_, ***X***_2_ and behaviour vectors ***b***_1_, ***b***_2_.
      2. Transform fMRI data into a PCA subspace; this allows for flexible model optimization by varying the PCA subspace size k. Perform Singular Value Decomposition (SVD) ***X***_i_ = ***U***_i_***Λ***_i_***V***_i_^T^ (i=1,2), where ***U***_i_ is a set of orthonormal image basis vectors, ***Λ***_i_ is a diagonal matrix of singular values, and ***V***_i_ is a set of orthonormal subject-weight vectors. ***X***_i_ is expressed in a reduced k-dimensional PCA space, by projecting onto the image bases ***U***_i_^(k)^ = [**u**_1_ **u**_2_ … **u**_k_], producing matrix ***Q***i^(k)^ = ***U***_i_^(k)T^***X***_i_.
      3. Perform PLS analysis on each ***Q***_i_^(k)^, by normalizing and centering subject scores of each PC-basis and subject behavioural scores, and obtain linear basis weights ***w***_i_ = ***Q***_i_^(k)^***b***_i_ that explained the most behavior variance in each split.
      4. Obtain predicted behavioural correlation by projecting split-2 data onto the split-1 PC-space, and then onto weight vector ***w***_1_, giving subject scores ***s*** = (***U***_1_^(k)T^***X***_2_)^T^***w***_1_. Compute the correlation *P*_behav_ = *corr*( ***s***, ***b***_2_ ), which measures how well split-1 data predicts behavioural correlations in independent split-2 data. Then repeat this process, projecting split-1 data onto the split-2 brain LV, and again measure correlation *P*_behav_.
      5. Measure reproducibility of the spatial brain LV by reconstructing the voxel map of brain regions most correlated with behavior for each split, ***e***_i_ = ***U***_i_^(k)^***w***_i_. Then compute the correlation between independent brain LV maps *R*_behav_= *corr*( ***e***_1_, ***e***_2_ ), which can be used to estimate *gSNR*_behav_ using Eqn. [S6]. We also estimate a reproducible Z-scored behavioural SPM using the procedure outlined in *Performance Metrics* section above.

We compute median *P*_behav_ and *gSNR_behav_*, and an average rSPMZ, across the 1000 resampling splits. This is done while varying the split-half PC subspace size from *k*=1 to *S/2*, and selecting *k* that minimizes the Euclidean distance from (*P*_behav_=1, *R*_behav_=1), similar to our pipeline optimization approach.
