# Supplementary material for: An Automated, Adaptive Framework for Optimizing Preprocessing Pipelines in Task-Based Functional MRI
Source: PLoS One. 2015 Jul 10;10(7):e0131520. doi: 10.1371/journal.pone.0131520 (PMC4498698; doi:10.1371/journal.pone.0131520)
Supplement: S5 Text — This procedure is used to detect subjects with activation patterns that are significant outliers, based on their influence in multivariate Principal Component space. This allows us to remove them prior to behavioural analyses, in order to improve the stability of results. (DOCX) [file pone.0131520.s011.docx]

**Text S5:** Identifying Outlier Subjects before Group Analysis

It is common in many fMRI studies to perform two-stage inferential testing. That is, we perform within-subjects analysis of the preprocessed fMRI data, followed by group-level testing of the resulting subject SPMs. Given relatively small sample sizes often collected for high-dimensional fMRI data, multivariate behavioural analyses tend to be highly sensitive to outlier subjects. We propose the following simple procedure for detection of multivariate outlier subjects, to be performed prior to analysis. It detects the degree of influence each subject has on the PCA decomposition of the data; subjects with extremely high influence are considered potential outliers in the dataset.

For *S* subject SPMs, concatenated in (*V* x S) data matrix ***X***:

1. On the full data matrix, perform a Singular Value Decomposition (SVD) of ***X*** = ***UΛV***^T^, where ***U*** is a set of orthonormal image basis vectors, ***Λ*** is a diagonal matrix of singular values, and ***V*** is a set of orthonormal subject-weight vectors. Record the variance-scaled eigenimage basis ***U**** = ***UΛ***.
2. For each subject s=1…S, remove this subject SPM from the matrix, giving reduced ***X***_-s_, and compute the SVD on this matrix, obtaining variance scaled eigenimage basis ***U****_-s_. Now, compute the RV coefficient between this and the full-data basis. This is a measure of multivariate subspace similarity, defined by:

$RV\left( \boldsymbol{X},\boldsymbol{Y} \right)=\frac{trace\left\{ (\boldsymbol{X}\boldsymbol{X}^{T})(\boldsymbol{Y}\boldsymbol{Y}^{T}) \right\}}{\sqrt{trace\left\{ {(\boldsymbol{X}\boldsymbol{X}^{T})}^{2} \right\}}\sqrt{trace\left\{ {(\boldsymbol{Y}\boldsymbol{Y}^{T})}^{2} \right\}}}$***

We record *RV* distance of subject s, defined ***d***(s) = 1 - *RV*( ***U***, ***U****_-s_ ). This is a measure of how much removing this subject alters the PCA subspace. A larger ***d***(s) value indicates greater influence on the overall subspace, indicating that this is an outlier point that dominates the covariance structure

1. We fit a Gamma probability distribution on the ***d*** values, by computing the maximum likelihood estimates of the distribution parameters. The Gamma model was used, as it forms a distribution over a set of random, strictly positive variables. We then identified subject volumes that are outliers at p<0.05.
2. These subject volumes are labeled as outliers, and discarded prior to second-level behavioural analyses.

This procedure minimizes the influence of outlier subjects on behavioural analyses.
